# Effects of low-intensity pulsed focal ultrasound-mediated delivery of endothelial progenitor-derived exosomes in tMCAo stroke

**DOI:** 10.3389/fneur.2025.1543133

**Published:** 2025-04-09

**Authors:** Ahmet Alptekin, Mohammad B. Khan, Mahrima Parvin, Hasanul Chowdhury, Sawaiz Kashif, Fowzia A. Selina, Anika Bushra, Justin Kelleher, Santu Ghosh, Dylan Williams, Emily Blumling, Roxan Ara, Asamoah Bosomtwi, Joseph A. Frank, Krishnan M. Dhandapani, Ali S. Arbab

**Affiliations:** ^1^Tumor Angiogenesis Laboratory, GCC, Department of Biochemistry and Molecular Biology, Medical College of Georgia, Augusta University, Augusta, GA, United States; ^2^Department of Neurology, Medical College of Georgia, Augusta University, Augusta, GA, United States; ^3^Department of Biostatistics, Medical College of Georgia, Augusta University, Augusta, GA, United States; ^4^Small Animal Imaging Core, GCC, Medical College of Georgia, Augusta University, Augusta, GA, United States; ^5^Laboratory of Diagnostic Radiology Research, Clinical Center, National Institutes of Health, Bethesda, MD, United States; ^6^Department of Neurosurgery, Medical College of Georgia, Augusta University, Augusta, GA, United States

**Keywords:** ischemic stroke, exosomes, low-intensity-pulsed focused ultrasound (LIPFUS), magnetic resonance imaging (MRI), single photon emission computed tomography (SPECT)

## Abstract

**Introduction:**

Exosomes from different sources have been used for therapeutic purposes to target stroke and other disorders. However, exosomes from endothelial progenitor cells (EPCs) have not been tested in any stroke model, and *in vivo* bio-distribution study is lacking. Targeted delivery of IV-administered exosomes has been a significant challenge. Delivery of exosomes to the brain is a daunting task, and a blood–brain barrier (BBB)-penetrable peptide is being considered. However, the next step in practical treatment will be delivering naïve (unmodified) exosomes to the stroke site without destroying host tissues or disrupting BBB, or the membranes of the delivery vehicles. Low-intensity-pulsed focused ultrasound (LIPFUS) is approved for clinical use in the musculoskeletal, transcranial brain, and physiotherapy clinics. The objectives of the proposed studies were to determine whether LIPFUS-mediated increased delivery of EPC-derived exosomes enhances stroke recovery and functional improvement in mice with transient middle cerebral artery occlusion (tMCAo) stroke.

**Methods:**

To enhance exosome delivery to the stroke area, we utilized LIPFUS. We evaluated stroke volume using MRI at different time points and conducted behavioral studies parallel to MRI to determine recovery. Ultimately, we studied brain tissue using immunohistochemistry to assess the extent of stroke and tissue regeneration.

**Results and Discussion:**

*In vivo*, imaging showed a higher accumulation of EPC exosomes following LIPFUS without any damage to the underlying brain tissues, increased leakage of albumin, or accumulation of CD45+ cells. Groups of mice (14–16 months old) were treated with Vehicle (PBS), LIPFUS only, EPC-exosomes only, and LIPFUS+EPC-exosomes. LIPFUS + EPC exosomes groups showed a significantly decreased stroke volume on day 7, decreased FluoroJade+ cells, and significantly higher numbers of neovascularization in and around the stroke areas compared to that of other groups.

## Introduction

Stroke is a significant cause of adult mortality and remains a leading contributor to adult disability. The majority of strokes are often ischemic (87%) resulting from a significant vascular occlusion due to a thromboembolic (TE) clot; therefore, the desired amount of stroke-salvaging drugs cannot be delivered to the core of the stroke (1, 2). In addition, the blood–brain barrier (BBB) is largely impermeable to most therapeutics. To date, IV tissue plasminogen activator (tPA) and/or endovascular thrombectomy (ET) are the only two Food and Drug Administration (FDA)-approved therapies to treat ischemic stroke. However, the recanalization of major vessels with IV-tPA/ET does not ensure adequate microvascular perfusion and recovery of tissue damage due to the associated risk of the “no-reflow” phenomenon that is exacerbated under stroke-related comorbidities such as aging, diabetes, or hypertension. Preclinical modeling using the transient middle cerebral artery occlusion (tMCAo) model, validated in both mice and rats, can replicate confirmed recanalization and is widely accepted in stroke research (3). Thrombectomy in patients, and tMCAo in animals, assures that the test drug or treatment reaches the brain after ischemia and reperfusion (4). Thus, effective therapy and a new way of delivering therapeutic agents that can be safe in a larger comorbid stroke population and remain usable in multiple settings with or without IV-tPA/ET even after the therapeutic window are greatly needed.

Stem cell (or progenitor cell) treatments have shown to be successful in various preclinical models for different disease treatments, including stroke. Our previous studies showed the effectiveness of endothelial progenitor cells (EPCs) and neural stem cells (NSCs) in stroke models, with both *in vivo* magnetic resonance imaging (MRI) and functional behavioral studies showing improvement in stroke recovery (5–9). We postulate that both types of cells exerted a paracrine effect through exosomes, which might carry materials from their parental cells (10–12). Exosomes can interact with cells by fusion with the plasma membrane and subsequent endocytosis and release of their cargo, consisting of proteins, soluble factors, lipids, DNAs, microRNAs (miRNAs), and RNAs (13–16). Leveraging exosomes to harness the therapeutic potential of stem and progenitor cells would overcome the challenge that cell-based treatments face in clinical settings.

Exosomes are 30–150 nm in size and contain certain tetraspanins, heat shock proteins, biogenesis-related proteins, membrane transport and fusion proteins, nucleic acids, and lipids (17–19). Due to their biocompatibility, low toxicity, immunogenicity, permeability (including through the **BBB**), stability in biological fluids, and ability to accumulate in the lesions with higher specificity (20–26), investigators have used exosomes in different disease conditions in the brain, such as stroke, traumatic brain injury, and tumors (22, 27–30). Investigators have used exosomes and miRNA-rich exosomes collected from mesenchymal stem cells (MSCs) to enhance stroke recovery and showed that miRNA promoted neural plasticity and functional recovery (31, 32). Our group recently reported NSC-derived exosomes’ effect on improving tissue and functional recovery in the murine TE stroke model (33). However, exosomes from EPCs, which play an important role in vascular regeneration, have not been tested in any stroke model, and an *in vivo* bio-distribution study is lacking. Our published studies showed a dramatic reduction in stroke injury when EPCs were administered 24 h following stroke (5). Based on these results, we anticipate that EPCs-derived exosomes will cease the progression of stroke lesions and improve post-stroke outcomes if delivered efficiently to the site of stroke injury.

Targeted delivery of IV-administered exosomes has been a great challenge. Modification of the exosome surface to carry different ligands or peptides has been tried to increase delivery to target tissues (34, 35); however, the overall results are not encouraging (36, 37). Delivery of exosomes to the brain is a daunting task, and a BBB-penetrable peptide is being considered (38, 39). However, a method that can deliver naïve (unmodified) exosomes to the site of stroke without destroying host tissues, disrupting BBB, or affecting the membranes of the delivery vehicles (such as exosomes) would be a significant breakthrough.

High-Intensity Pulsed Focused ultrasound (HIPFUS) is being investigated to enhance permeability and retention (EPR) of nanoparticles/gene/plasmid/vectors to the sites of interest (40–45) but has been shown to cause widespread damage to local tissues including the brain (46). The basic mechanism behind HIPFUS’s effect is primarily through mechanical forces (acoustic radiation and acoustic cavitation) that increase the permeability of the vasculature, resulting in leakage of circulating nanoparticles, plasmids, or vectors into targeted sites and EPR (47–49). Low-intensity-pulsed ultrasound is approved for clinical use in musculoskeletal, central nervous system, and physiotherapy clinics. We have previously demonstrated that low-intensity-pulsed focused ultrasound (**LIPFUS**) increases the delivery of intravenously administered exosomes to stroke areas without causing damage to the brain (50). The objectives of the proposed studies are to determine whether LIPFUS-mediated increased delivery of EPC exosomes enhances stroke recovery and functional improvement in mice with tMCAo stroke.

In this study, we utilized EPC-derived exosomes for stroke treatment. To enhance exosome delivery to the stroke area, we employed LIPFUS without an ultrasound contrast agent microbubble/nanobubble infusion, which might temporarily disrupt the BBB, as described in our previous study (50). We assessed stroke volume using MRI at different time points and conducted behavioral studies parallel to MRI to evaluate recovery. In the end, we examined the brain tissue using immunohistochemistry studies to evaluate the extent of stroke and tissue regeneration. We found that EPC-derived exosome treatment reduced stroke volume 1 week after the stroke onset.

## Materials and methods

### Ethics statement

This study adhered to the guidelines and regulations set forth by the National Institutes of Health (NIH). Experimental protocols were approved by the Institutional Animal Care and Use Committee (IACUC) of Augusta University (protocol #2014-0625). Animal housing conditions included a standard environment with a 12 h light–dark cycle, and animals had unrestricted access to food and water. Stringent measures were implemented to minimize any potential distress experienced by the animals. After CO_2_ overdose and cervical dislocation, animals underwent trans-cardiac perfusion with buffered saline and paraformaldehyde, followed by cardiac transaction and bilateral pneumothorax as a secondary assurance of death. This method is consistent with the recommendations of the Panel on Euthanasia of the American Veterinary Medical Association (AVMA) and allows for the best preservation of tissue architecture for in vitro assessment.

All mice (14–16 months old, 30–35 gm) were randomized. Studies were conducted in multiple batches and each batch had animals for experimental as well as corresponding control groups. Personnel blinded to the various groups analyzed data. The animals were of the same age and weight and had similar baseline behavior. The study is reported according to the ARRIVE guidelines.

### EPC collection

Investigators, including our team, have reported the collection of EPCs based on Sca-1 (stem cell antigen-1) or c-Kit (CD117) positive markers (51–56). The cells were grown with EPC media as described in our previous studies (5, 57). These cells are shown to have all the characteristics of EPC concerning cellular markers and functions (51, 56). In this study, EPCs were collected from the bone marrow of young (10–12 weeks old) mice by positive selection of c-kit (+) cells. Briefly, mice were sacrificed using CO_2_, and the spine and long bones were collected sterilely. Cells were extracted by crushing the bones, flushing them with PBS (pH ~7.4), and filtering the cell suspension through a 70-micron cell strainer (22-363-548, Fisherbrand). Nucleated cells were then separated from erythrocytes using Lymphocyte Separation Medium (25-072-CV, Corning). EPCs were isolated according to the manufacturer’s protocol (8802-6838-74 MagniSort™ Mouse CD117 (c-Kit) Positive Selection Kit, Invitrogen) and were collected and grown in EPC media (CellGenix GMP SCGM) supplemented with growth factors for EPCs. EPC cells were cultured according to our previously published techniques for collecting and propagating EPCs (51, 57) using exosome-free media.

### Exosome isolation

Exosomes were isolated according to our previously reported protocol (11) and stored at −80° until use. Briefly, culture media from EPC were collected, cell debris was removed by centrifugation, and media was dialyzed using Macrosep Advance Centrifugal Device to remove all molecules below 100 kDa in molecular weight. The concentrated media was then diluted with PBS and ultracentrifuged for 70 min at 137,000 g, at 4°C. Exosomes were collected from the bottom of the tube and stored at −80°C until use. The size and number of exosomes were determined by ZetaView (Particle Matrix, GmbH). We have already reported the size of exosomes in our previous publication (50). Exosomes were administered intravenously (IV) to the stroke-bearing animals with or without LIPFUS within 24 h after surgery.

### Stroke surgery

**Figure 1:**
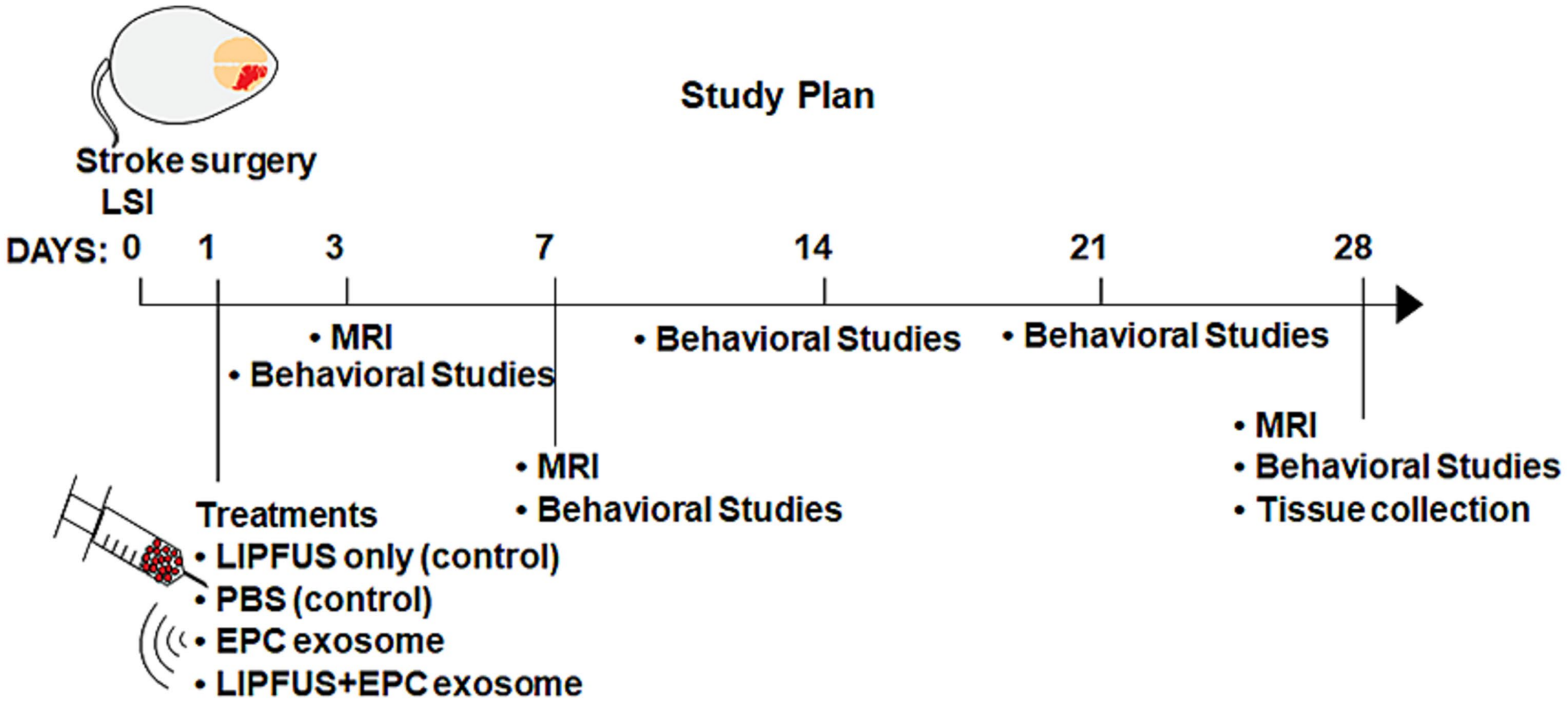
Making of strokes, treatment, and neurobehavioral study plans.

Aged mice (both males and females, 14–16 months old) were anesthetized using isoflurane, and the right common carotid artery, right external carotid artery (ECA), and right internal carotid artery (ICA) were exposed (58). A monofilament suture (Doccol Corp) was introduced through the right ECA into the ICA and the middle cerebral artery until resistance was felt and placed for 60 min. Cerebral blood flow was determined by laser speckle imaging (LSI), and the filament was gently withdrawn after 60 min. Exosome treatments and LIPFUS were given one day after the surgery, and MRI images were acquired on 3 days, 7 days, and 28 days post-surgery. The study plan is illustrated in Figure 1.

### Low-intensity-pulsed focused ultrasound

The RK-50 (FUS Instruments Inc., Canada) is a standalone preclinical focused ultrasound system designed for high throughput blood–brain barrier experiments. Atlas-based targeting combined with an optional multi-modality high field insert allows the system to be used with or without magnetic resonance imaging (MRI) or X-ray computed tomography (CT) guidance. The system is based upon a flexible cross-platform animal handling system that simplifies handling and enhances efficiency. The instrument has a three-axis motorized positioning system and an accurate stereotaxic-guided targeting system that uses rodent brain atlases (mouse & rat). It has a calibrated spherically focused ultrasound transducer (typically 1.47 MHz) with a maximum radio frequency power of 15 watts and includes animal restraint and inhalant anesthetic fixtures. The diameter of the transducer is 35 mm, the F factor is 0.7, the focal areas are (FWHM of the pressure field) 1 mm laterally and 6 mm axially, and the focal depth is 24 mm from the surface of the transducer. It also has custom-written software for atlas registration and treatment planning capability to deliver single or multi-point acoustic exposures (continuous or pulsed).

Our previously published report showed the optimized parameters of LIPFUS for the delivery of exosomes to the site of stroke (50). A fixed acoustic frequency of 1.47 MHz for deeper penetration with minimal thermal effect using a focal transducer (wavelength of 1.047 mm) was used. LIPFUS was applied to 6–10 points (minimum 1 mm separation) based on the size of the stroke with a 1% duty cycle (burst duration of 10 milliseconds (ms), repetition period 1,000 ms) for 90 bursts. 2 Mega Pascal (MPa) peak negative pressure (acoustic power) to the tissue without an ultrasound contrast agent was applied.

### SPECT–CT study

To determine the accumulation of IV administered EPC derived exosomes at the sites of stroke with (*n* = 4) or without LIPFUS (*n* = 4), collected exosomes were labeled with Iodine-131 (I-131) using Pierce iodination beads as per the protocol described by the manufacturer (ThermoFisher, United States). In short, based on the initial number of exosomes we allocated I-131 in a small volume of normal saline (for the final activity of ~100 μCi tagged with 3 billion exosomes) in a reaction tube. 2–3 beads were added to the solution and incubated for 5 min. Then the desired number of exosomes was added to the solution in a small volume of saline (~0.5 mL) and the reaction was continued for 30–40 min. The reaction was stopped by separating the beads from the solution. To get rid of the free I-131, 2–3 mL normal saline was added to the solution and the solution was filtered through a 100 k centrifugal filter (Pall Corporation, United States). Labeling efficiency and binding affinity were determined using thin-layered chromatography and serum challenge as per our previously published method (11). Twenty-four hours after stroke and following LIPFUS I-131 labeled exosomes were administered IV and SPECT–CT images were obtained at 3 h and 24 h. After the intravenous injection of 100 ± 25 μCi of I-131-labeled exosomes in 100–200 μL into the tail vein of the mice, whole body, as well as focal (brain) SPECT images, were acquired using a dedicated 4-headed NanoScan, high-sensitivity microSPECT/CT 4R (Mediso, Boston, MA, United States) fitted with high-resolution multi-pinhole (total 100) collimators. The microSPECT has a wide range of energy capabilities from 20 to 600 keV, with a spatial resolution of 275 μm. The images were obtained using 60 projections with 30–60 s/projection, with a medium field of view. Attenuation was corrected using concurrent CT images, and then the images were reconstructed with low iteration and low filtered back projection. During the acquisition of images, subjects were under anesthesia with their body temperature and breathing monitored. Randomly selected separate groups of animals bearing stroke were used for SPECT–CT studies. All animals were euthanized following the last scans.

### Magnetic resonance imaging

All MRI experiments were conducted using a 7 Tesla horizontal magnet with a clear bore diameter of 20 cm, interfaced to a Bruker Avance console with actively shielded gradients. Animals underwent the following magnetic resonance (MR) imaging: (1) Multislice Multi-echo T2-weighted images, to create corresponding T2 maps to determine edema, ventricular, and stroke volumes; (2) Arterial spin labeling (ASL) imaging based on the Flow-sensitive Alternating Inversion Recovery (FAIR) technique, to determine the cerebral blood flow; (3) Diffusion Tensor Imagining (DTI), to determine the white matter tracts, changes in the fractional anisotropy (FA), tensor weighted images (TWI), and tensor trace (TT). TWI is like that of a diffusion-weighted image (DWI), and TT is like the apparent diffusion coefficient (ADC). (4) Modified Driven Equilibrium Fourier Transform (MDEFT) to determine the white matter tracts. This is like that of T1-weighted imaging with an inversion pulse to suppress water signals facilitating the easy identification of white matter. We have obtained MRI on days 3, 7, and 28 days following stroke surgeries. On day 3, we acquired T2 images to create maps to determine the stroke and ventricular volumes. These were regarded as the baseline and all subsequent measurements were normalized to day 3 values. We also used day 3 MRI for different parameters. MRI obtained on days 7 and 28 were used to measure stroke volume recovery, relative cerebral blood flow (rCBF), and measurement of different diffusion parameters and well as white matter tracts. It is known that delayed MRI (day 28) will not be helpful in measuring stroke volume; however, we used it for analyzing rCBF and white matter tracts and fractional anisotropy.

An appropriate state of anesthesia was obtained with isoflurane (2.5% for induction, 0.7 to 1.5% for maintenance in a 2:1 mixture of N_2_: O_2_). The anesthetized mice were imaged using a horizontal 7 Tesla BioSpec MRI spectrometer (Brucker Instruments, Billerica, MA) equipped with a 12 cm self-shielded gradient set (45 gauss/cm max). The radiofrequency (rf) pulses were applied using a standard transmit/receive (Tx/Rx) volume coil (72 mm I.d.), actively decoupled from the four-channel Bruker quadrature receive coil positioned over the centerline of the animal skull. Stereotaxic ear bars were used to minimize movement during the imaging procedure. Animal temperature was maintained at 37.8°C using a recirculating water bath. After positioning using a tri-planar FLASH sequence, MR studies were performed as follows: (1) T2-mapping sequence (2D multi-slice, multi-echo (MSME) sequence, TE = 10 to 150 ms, TR = 5,000 ms, FOV = 19.2 mm, 0.5 mm thick slice, 128×128 matrix, # of averages = 1, 30 slices), (2) DTI (EPI) images (TE/TR = 27/3000 ms; # of Averages = 1; Echo train length = 60, b value 0 and 1,000; 30 directions; Acq. Matrix = 96×96; 30 slices; slice thickness = 0.5 mm; FOV = 16.8.0× 16.8 mm; Partial Fourier Acceleration: 1.25), (3) ASL images (FAIR-RARE, Teeff = 46 ms, TR = 10,000 ms, inversion time = 1,400 ms, number of average = 20, 128×128 matrix, 2.4 mm slice thickness, FOV = 19.2 mm, 3 slices), (4) corresponding T1 maps (for quantification of rCBF) were created using standard T1 sequences with 5 different TR (5,500 to 326 ms, 2.4 mm thick), (5) T2 FLAIR sequence (RARE-IR, TI = 2,500, TR = 10,000, TE = 22, RARE factor = 8 FOV = 19.2 mm, 128×128 matrix, 0.5 mm slice thickness, 30 slices, number of average = 2), and (6) MDEFT to determine the white matter tracts (TI = 1,100, TR = 3,000, TE = 3.5, FA = 15, FOV = 19.2 mm, 128×128 matrix, 0.5 mm slice thickness, 30 slices, number of averages = 2).

### MRI analysis

T2 maps of MRI images were analyzed in Mango, Multi-image Analysis Graphical User Interface (v4.1, Research Imaging Institute, UTHSCSA). Region of Interest (ROI) was selected by applying a threshold with a minimum value of 58–65 ms. Once the stroke area was visualized with an ROI mask, other regions (ventricles and outside of the brain) were excluded. Marked ROI on stroke area was saved. Then T2 map was opened in ITK-SNAP (v3.6.0), the ROI of stroke was uploaded as segmentation, and volume was calculated as mm^3^. The same steps are repeated to calculate ventricle volumes, except ROI to detect ventricles was set at 90 ms. ROIs of left and right ventricles were determined, and volumes were calculated in ITK-SNAP separately. Statistical significance was calculated using two-way ANOVA in GraphPad Prism (v9.2.0), and the graphs detailing the statistical significance were generated.

Determination of the volume of white matter was done using MDEFT images. In this imaging sequence, an inversion pulse is applied to attenuate signals from free water (like that of T2 FLAIR), but the images look like T1-weighted images. All images were analyzed by investigators who were blinded to the treatments. ImageJ (NIH) image analysis software (ImageJ v1.52a) was used. After proper thresholding, the volume of white matter (both left and right hemispheres separately) was determined by making irregular ROI to encompass the white matter. The right-to-left ratio (volume of right hemisphere/volume of left hemisphere) was determined and compared across different groups of animals as well as with immunohistochemistry images. Day 28 MDEFT images were also used to determine the volume of the right and left hemispheres. Five MRI sections (at the level of Bregma plus 2 sections anterior and 2 sections posterior) were used to measure the volume and the right-to-left hemisphere ratio was calculated and compared among the groups.

Relative cerebral blood flow (rCBF) was determined using FAIR (ASL) maps. This was determined by making ROI on the right hemisphere (encompassing the stroke area) and then the corresponding left hemisphere. The right-to-left ratio (rCBF right hemisphere/ rCBF left hemisphere) was determined and compared among different groups of animals. Corresponding slices that contained strokes were used to measure TWI and TT. Identical ROI was used both on the right and left hemispheres and the right-to-left ratio was determined.

We have not noticed any new high signal intensity areas in the periventricular regions on day 7 and day 28 FLAIR images, and we have not quantitatively analyzed the data from FLAIR images.

### Behavioral studies

To assess neurobehavioral recovery in mice after stroke surgery, several neurobehavioral tests were conducted in both the control and treatment groups. We used the Bederson score (0–3) (59), and the Corner test (60) on days 3, 7, 14, 21, and 28 following surgeries.

### Luxol Fast Blue staining

To determine the changes in the white matter tracts, Luxol Fast Blue (LFB) staining was used. Formalin-fixed paraffin-embedded (FFPE) tissues were de-paraffinized by serial incubation of xylene, 100% ethanol, and 95% ethanol. Then slides were incubated in LFB solution (0.1 g LFB (212,170,250, Acros Organics), 100 mL 95% ethanol, 0.5 mL 10% acetic acid) at 56°C overnight. Slides were rinsed with 95% ethanol and water, then differentiated in Lithium carbonate solution (0.25 gm lithium carbonate, 500 mL distilled water). After incubation with 70% ethanol, slides were washed and counterstained with cresyl violet solution for 5 min, rinsed with water, dehydrated with 95% ethanol and xylene incubations, and coverslip mounted. The LFB+ area was determined in the stroke and contralateral hemispheres of each animal. The right-to-left ratio (LFB+ area in right hemisphere/ LFB+ area in left hemisphere) was determined and compared across different groups of animals.

### Fluoro-Jade C staining

To determine the level of degenerating neurons, Fluoro-Jade C staining was performed. FFPE tissue slides were de-paraffinized and stained with Fluoro-Jade C (TR-100-FJ, Biosensis), according to the manufacturer’s protocol. Briefly, slides were serially incubated with potassium permanganate, Fluoro-Jade C solution, and DAPI. Then, in a fluorescent microscope, we acquired microscopic images of Fluoro-Jade C slides in the green channel.

### Hematoxylin eosin staining

FFPE slides were de-paraffinized, and serially incubated in hematoxylin, acid alcohol (1% HCl in 70% ethanol), water, ammonia water (1 mL NH_4_OH in 1 L water), water, and eosin Y. After rinsing with water, the slides were dehydrated in ethanol and xylene and mounted with a coverslip.

### Immunohistochemistry

#### Antigen retrieval

Deparaffinized slides were incubated in citrate buffer (10 mM sodium citrate, 0.05% Tween 20, pH: 6.0) at 92°C for 40 min and cooled to room temperature for another 30 min. They were then rinsed with water and PBS before blocking.

Standard immunofluorescent staining was performed to determine the distribution of white matter tracts (using anti-myeline basic protein (MBP)), reactive astrocytes (anti-GFAP), neurons (anti-NeuN), and endothelial lining (Tomato lectin). Fluorescent intensity (indicating MBP density) and MBP+ area in the stroke and contralateral hemispheres were determined. The right-to-left ratio (MBP+ area in the right hemisphere/ MBP+ area in the left hemisphere) was determined and compared with different groups of animals. Similarly, fluorescent intensity, as well as the area (number) of GFAP cells, were determined in both the stroke and contralateral hemispheres. The right to left ration was determined and compared with different groups of animals. We also determined the number of tomato lectin-positive areas, and NeuN+ cells in the stroke and contralateral hemispheres and compared among groups. All these analyses were done using ImageJ.

To determine the effect of LIPFUS on the leakage of BBB and accumulation of inflammatory cells (CD45+ and CD68+) in the stroke areas, groups of animals (with or without LIPFUS) were euthanized 24 h after LIPFUS (48 h after stroke) and accumulation of albumin and CD45+ or CD68+ cells were determined by standard immunofluorescent staining. Albumin is known to be present outside blood vessels once BBB leakage is significant and sustained (45, 61). Our previous publication also showed albumin as a sign of BBB leakage (50).

### Statistical analysis

To examine differences in MRI and neurobehavioral outcome measures between LIPFUS (yes or no), and exosomes (yes or no) over time, repeated measures mixed models followed by post-hoc tests were used. Different correlation structures between measurement times were investigated, including unstructured, compound symmetric, and autoregressive. An alpha level of 0.05 was considered significant. All data was expressed in mean ± SEM (standard error of the mean). We acknowledge that the final group sizes were reduced from our initial target due to unexpected mortality, technical issues, and resource limitations. Despite the imbalanced sample sizes, we applied appropriate statistical methods, such as mixed-effects models, which are robust to unequal group sizes. While the smaller group sizes may reduce overall power, the effect sizes observed were substantial, supporting the biological relevance of our findings despite variability in sample numbers.

## Results

### Isolation and characterization of endothelial progenitor cell-derived exosomes for stroke treatment

To utilize stroke treatment, we isolated EPCs from mouse bone marrow following selection with cKit antibody and cultured them in exosome-depleted EPC media in normoxic conditions. After 2 days of culture, the medium was collected, and exosomes were isolated using size exclusion filters and ultracentrifugation. We determined exosome concentration, and size distribution by ZetaView, which was previously published (50). The average size was 97.2 ± 39.8 nm. After EPC exosome treatment, there were no observable side effects or increase in mortality, indicating the safety of exosome treatment. We used a membrane cytokine array to determine the cytokine profile and compare the values with exosomes collected from other cell types from mice. The results are already shown in our previous publication (11).

### SPECT–CT study of EPC-exosome accumulation in stroke sites

**Figure 2:**
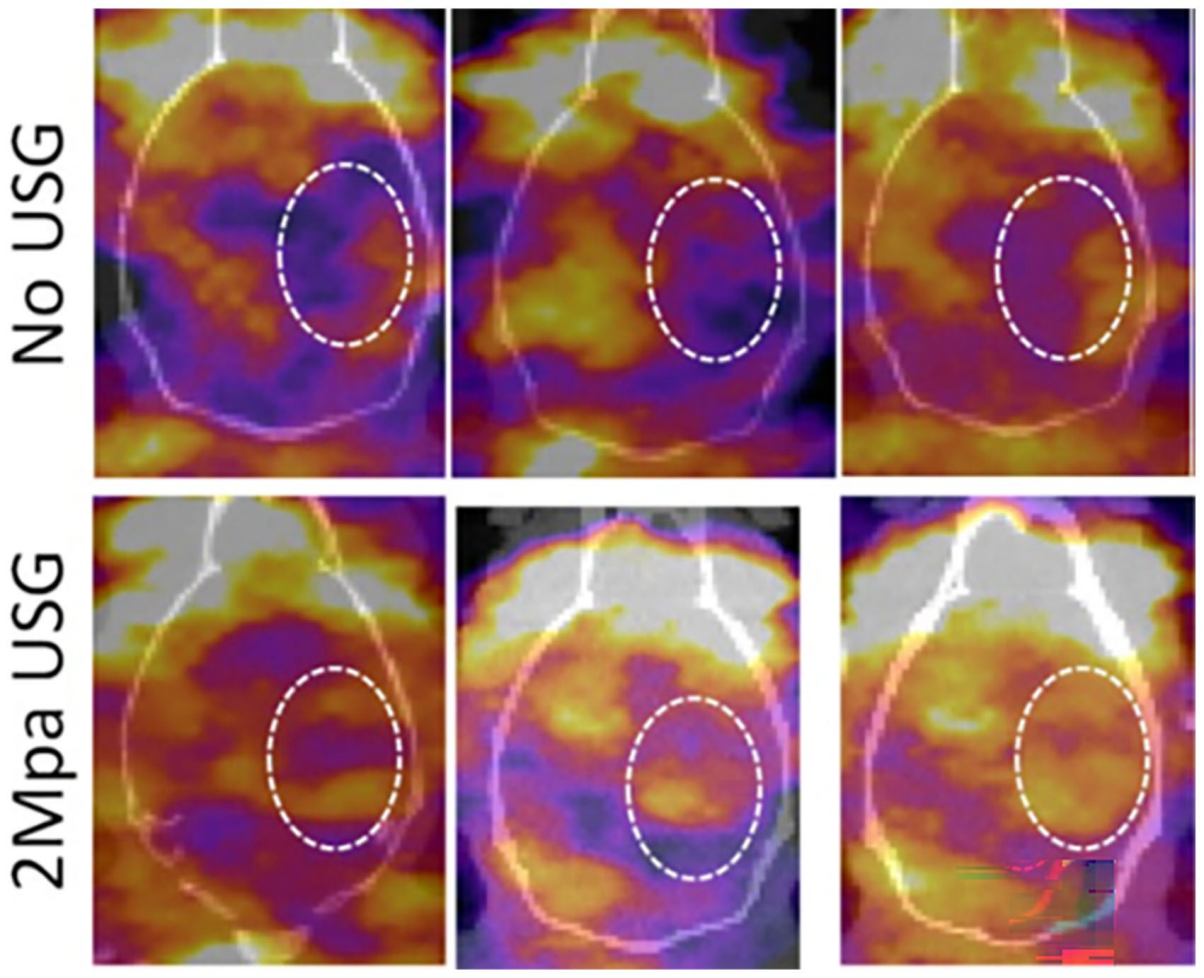
Representative SPECT images from three animals (each group) following IV administration of I-131 tagged exosomes with or without LIPFUS. Note the higher accumulation of I-131-tagged exosomes in the stroke area in animals that received LIPFUS (lower panel, inside dotted circles). USG = LIPFUS.

To determine the accumulation of EPC-derived exosomes to the sites of stroke with or without LIPFUS, radioisotope (I-131) labeled exosomes were IV administered, and SPECT–CT images were obtained at 3 h and 24 h following administration of exosomes. The SPECT–CT images clearly showed higher accumulation at the site of stroke, treated with exosomes and LIPFUS (Figure 2).

### Serial MR imaging shows that EPC exosome treatment significantly decreases stroke volume after one week

We performed stroke surgery on 49 older (14–16 months old) mice. Laser Speckle Imaging during the procedure confirmed circulation occlusion in all 49 mice (Supplementary Figure 1, Laser Speckle Images). 15 mice died before completing the study, and 12 mice did not have a stroke on day 3 MRI and were excluded from the study. A total of 22 mice (vehicle control 9 mice, LIPFUS control 3 mice, EPC exosome treatment 4 mice, EPC exosome and LIPFUS treatment 6 mice) completed the study.

**Figure 3:**
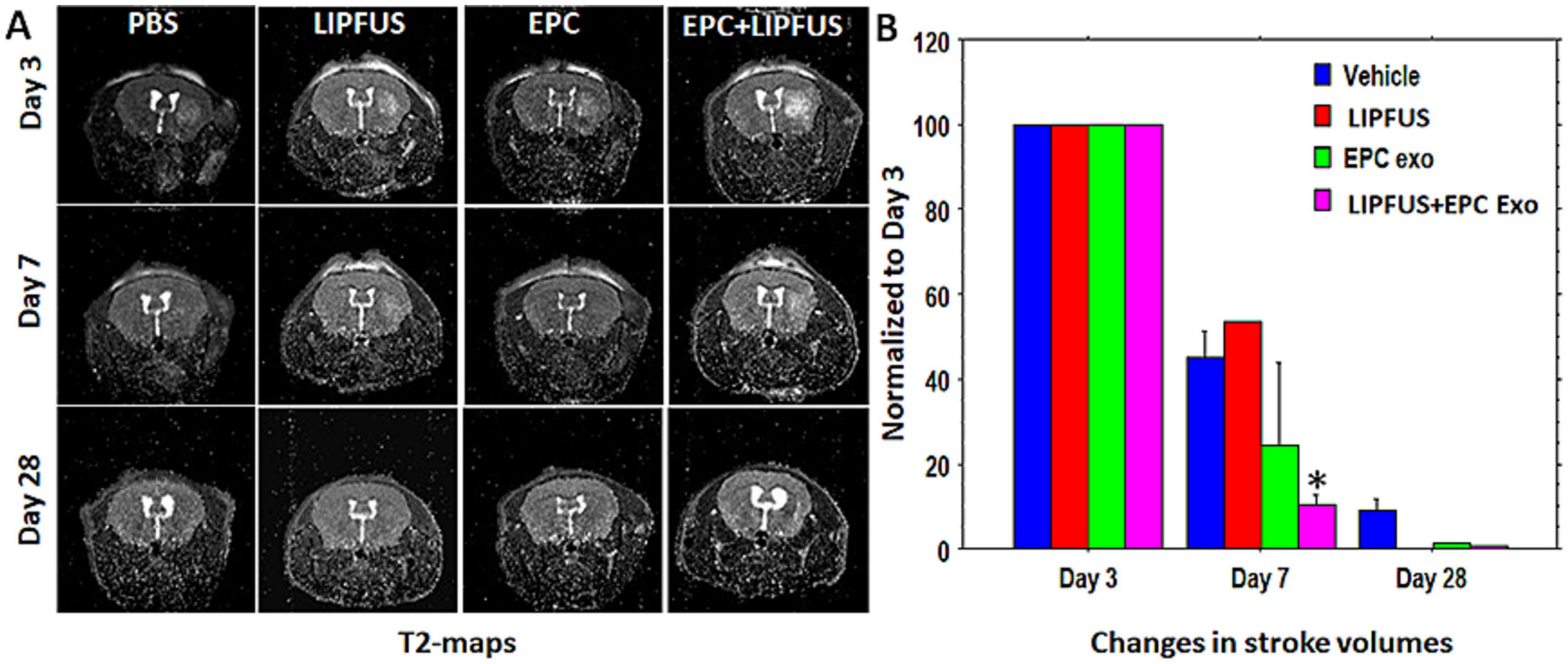
T2-maps and stroke volumes: **(A)** T2 maps show the area of stroke on days 3, 7, and 28. All data were normalized to the corresponding stoke volume on day 3. **(B)** There was higher rate (borderline significance) of stroke resolution (decreased stroke volume) observed in groups treated with LIPFUS+EPC exosomes (*p* = 0.08) Stroke volume measured on day 28 was not useful as a detectable high signal intensity area was not observed.

**Figure 4:**
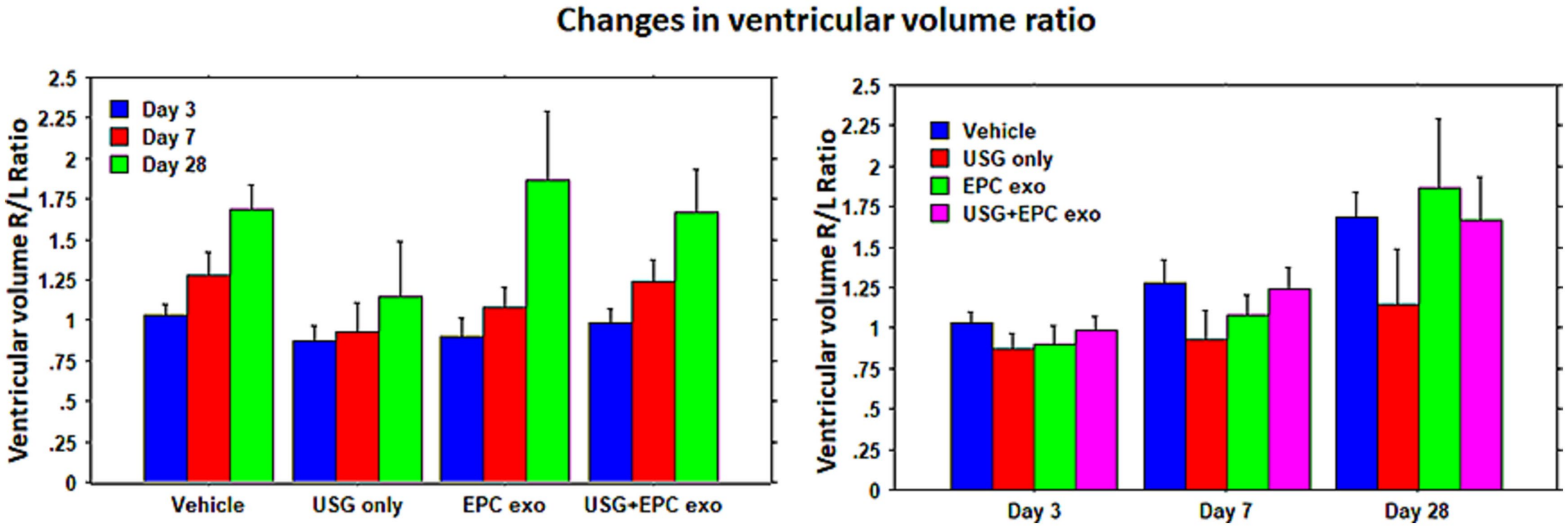
Ventricular volume: lateral ventricular volume was measured using T2-map images. The ratio of right lateral ventricular to left lateral ventricular volumes was determined. Although there was a higher resolution of stroke in animals treated with LIPFUS+EPC exosomes, there were no significant differences in the ventricular volume among the treated groups. USG = LIPFUS.

To evaluate stroke volume in response to treatment, we employed MRI imaging. On day 3 of stroke surgery, we obtained an MRI, which was used as the baseline to compare stroke volume with treatment. MRI could not be obtained before 3 days, because we observed that mice were not stable after stroke surgery, and the MRI in the first 2 days of the surgery posed a high risk for death during or immediately after the MRI. Therefore, on day 3, we acquired T2 images to create maps to determine the stroke and ventricular volumes. These were regarded as the baseline and all subsequent measurements were normalized to Day 3 values. MRI could not determine the stroke volumes clearly in all animals on day 28; therefore, our analysis was concentrated on day 7 data. Compared to all groups, significantly (*p* = 0.0176) lower stroke volume was observed in animals treated with LIPFUS followed by IV administration of EPC exosomes (Figure 3). There was also a smaller stroke volume observed in the EPC exosome-only treated group but did not achieve a significant difference. When comparing the ratio of right to left ventricular volume among the groups, there were no significant differences observed. All groups showed a gradual increase in right to left ventricular volume ratio indicating a gradual shrinking of right brain parenchyma following stroke in the right hemisphere (Figure 4).

### Cerebral blood flow and endothelial cells post-stroke

**Figure 5:**
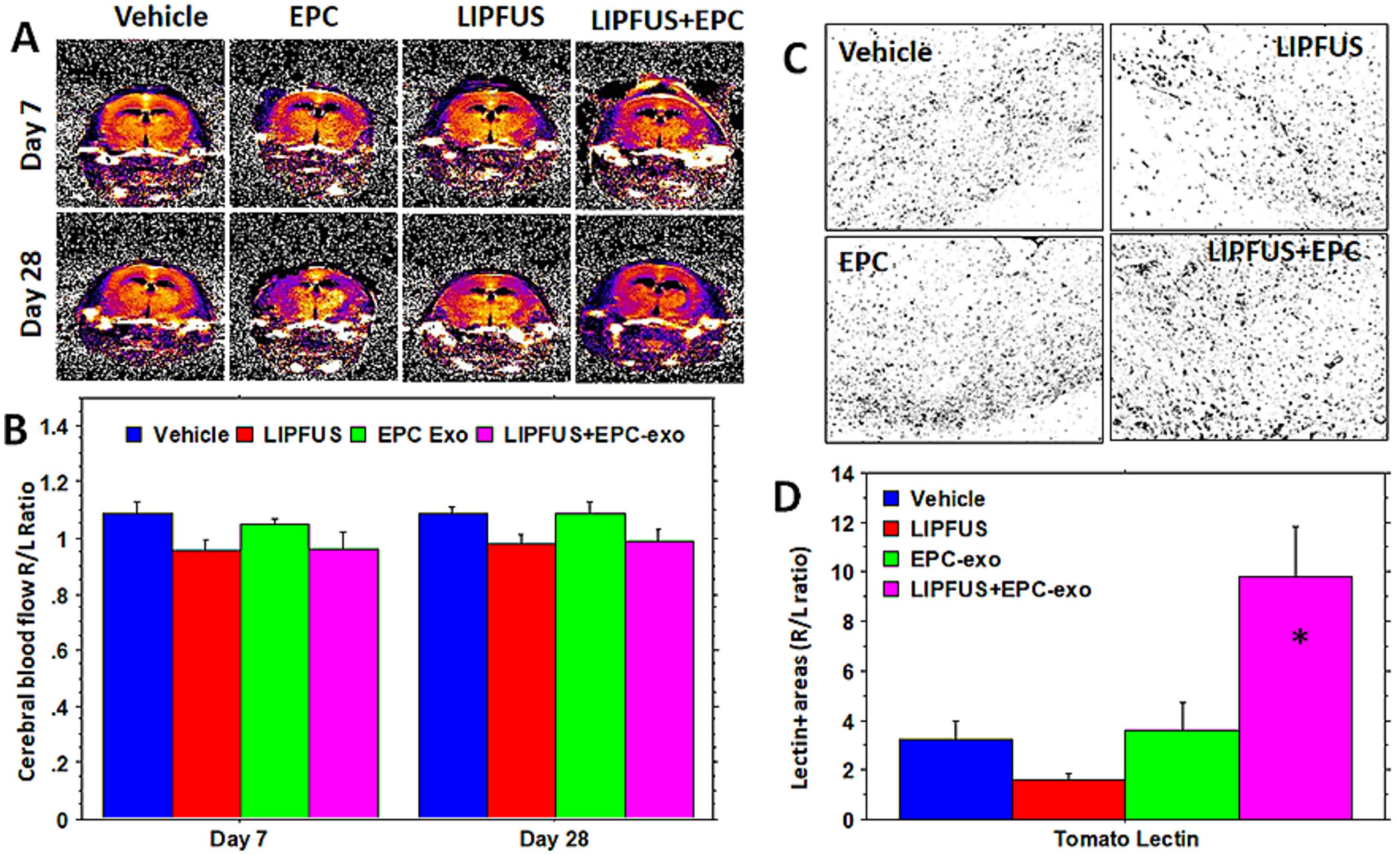
Cerebral blood flow and density of endothelial cells: arterial spin labeling technique shows similar cerebral blood flow ratio (right/left hemisphere) in the stroke areas and there is no significant difference among the treated groups **(A,B)**. However, when the density of neo-blood vessels is determined by tomato lectin staining **(C,D)**, there is a significantly higher density of tomato lectin-positive cells seen in and around the stroke areas in LIPFUS+EPC exosome-treated groups indicating a high number of blood vessel formation. This discrepancy could be due to less sensitivity of the MRI technique or lack of functional blood vessels.

To measure the improvement of blood supply we used arterial spin labeling (ASL, flow-sensitive alternating inversion recovery (FAIR)) sequences, and the flow was compared with the corresponding contralateral left hemisphere (ratio between right to left CBF). To our surprise, the LIPFUS+EPC exosomes group showed no changes of rCBF on days 7 and 28 compared to that of vehicle-treated animals (Figures 5A,B) although LIPFUS+EPC exosomes-treated animals showed comparatively significant resolution of stroke volume (decreased stroke volume) on day 7 (Figure 3). However, when we analyzed the Tomato lectin-positive areas (endothelial positive areas) in the sections from the stroke brains using ImageJ thresholding (masking), the LIPFUS+EPC exosomes group showed a significantly higher (*p* < 0.01) number of lectin-positive areas around the stroke sites (Figures 5C,D). This discrepancy could be due to the lack of establishment of functional channels (functional blood vessels) following the proliferation of endothelial cells at the site of stroke and the administration of LIPFUS and EPC exosomes.

Supplementary Figure 2 shows no increased extravasation of albumin around the blood vessels, and an increased number of CD45+ and CD68+ cells in the stroke areas following LIPFUS indicating no effect following transient BBB opening.

### MR imaging and histochemical analysis of white matter

**Figure 6:**
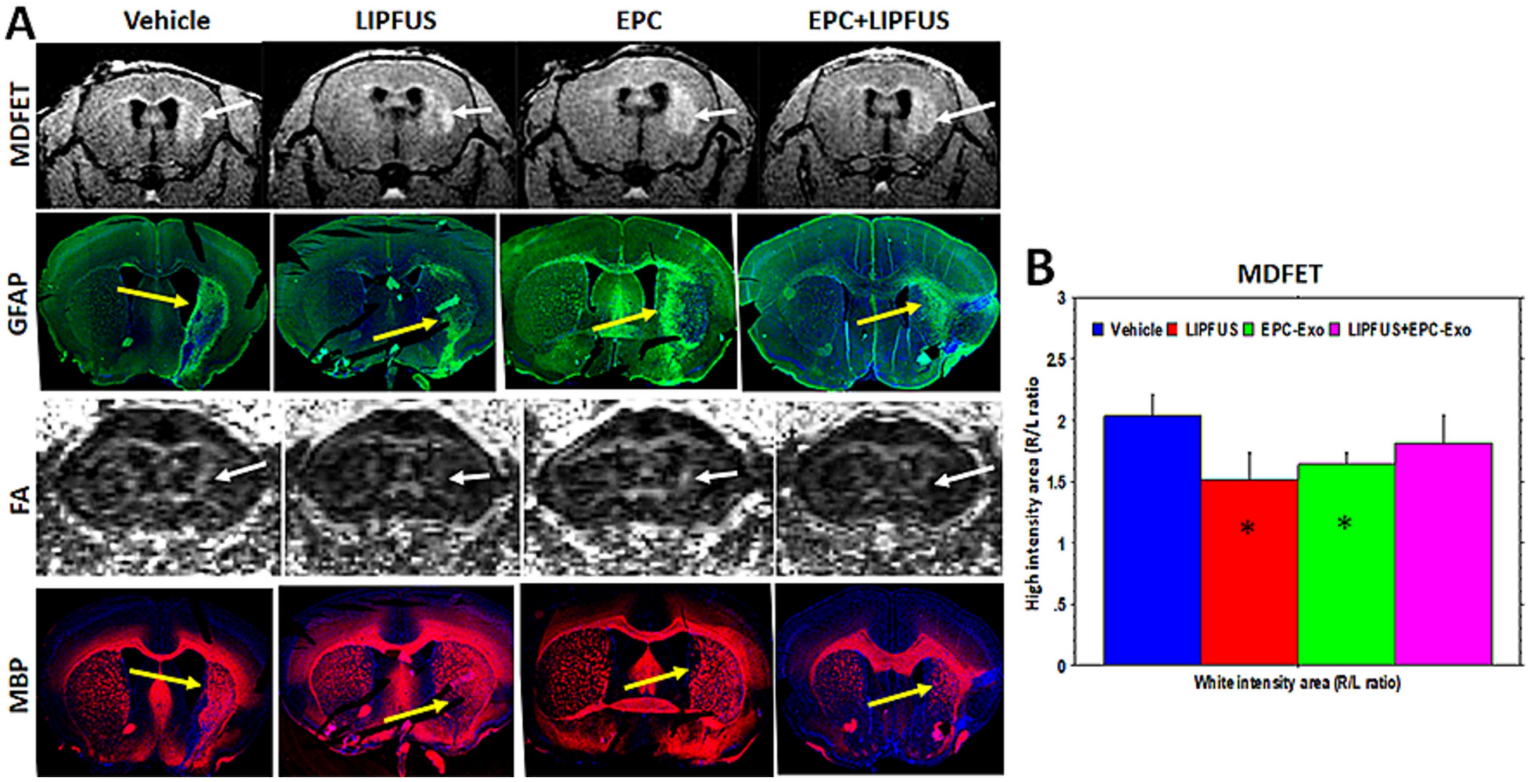
Astrogliosis and white matter tracts: **(A)** MRI images (MDFET) show large high signal intensity areas along the white matter tracts in the stroke hemispheres (white arrows). **(B)** There are significant differences (* *p* < 0.05) in the ratio of high signal intensity areas among the treatment groups. GFAP staining shows a higher number of activated astrocytes in the corresponding areas indicating astrogliosis (yellow arrows). However, DTI images (FA) show narrower white matter tracts, which deviate towards the midline and away from the astrogliosis (white arrows). MBP staining also indicates narrower white matter tracts; however, some of the smaller white matter tracts away from corpus callosum superimpose with that of astrogliosis (GFAP vs. MBP, multi-fluorescent IHC was performed on the same section).

To determine the white matter tracts, we employed two different MRI protocols. We used DTI sequences to determine the fractional anisotropy (FA) as well as MDEFT sequences to determine the white matter. We have noticed larger high signal intensity areas at the stroke sites on MDEFT compared to that of fractional anisotropy (FA) images observed on DTI sequences (Figure 6, white arrows). When the GFAP and MBP immunohistochemical images were analyzed, the high signal intensity areas seen on MDEFT images correspond with GFAP staining of activated astrocytes (Figure 6, yellow arrows). However, we did not find any enlarged MBP or LFB areas. Rather, both MBP and LFB staining showed decreased areas at the sites of strokes (yellow arrows), indicating loss of white matter tracts. It is also interesting to note that FA images show deviation of the white matter tracts towards the midline (Figure 6, white arrows). We also measured the volume of both brain hemispheres using MDEFT images (excluding the ventricles). Supplementary Figure 3 also shows the ratio of right to left hemisphere volume. PBS-treated animals showed the lowest ratio, on the other hand, EPC exosomes treated showed a significantly higher ratio on day 7 than that of PBS or LIPFUS-only or LIPFUS plus EPC exosomes treated animals indicating less loss of brain parenchyma.

### Assessment of additional MRI metrics

**Figure 7:**
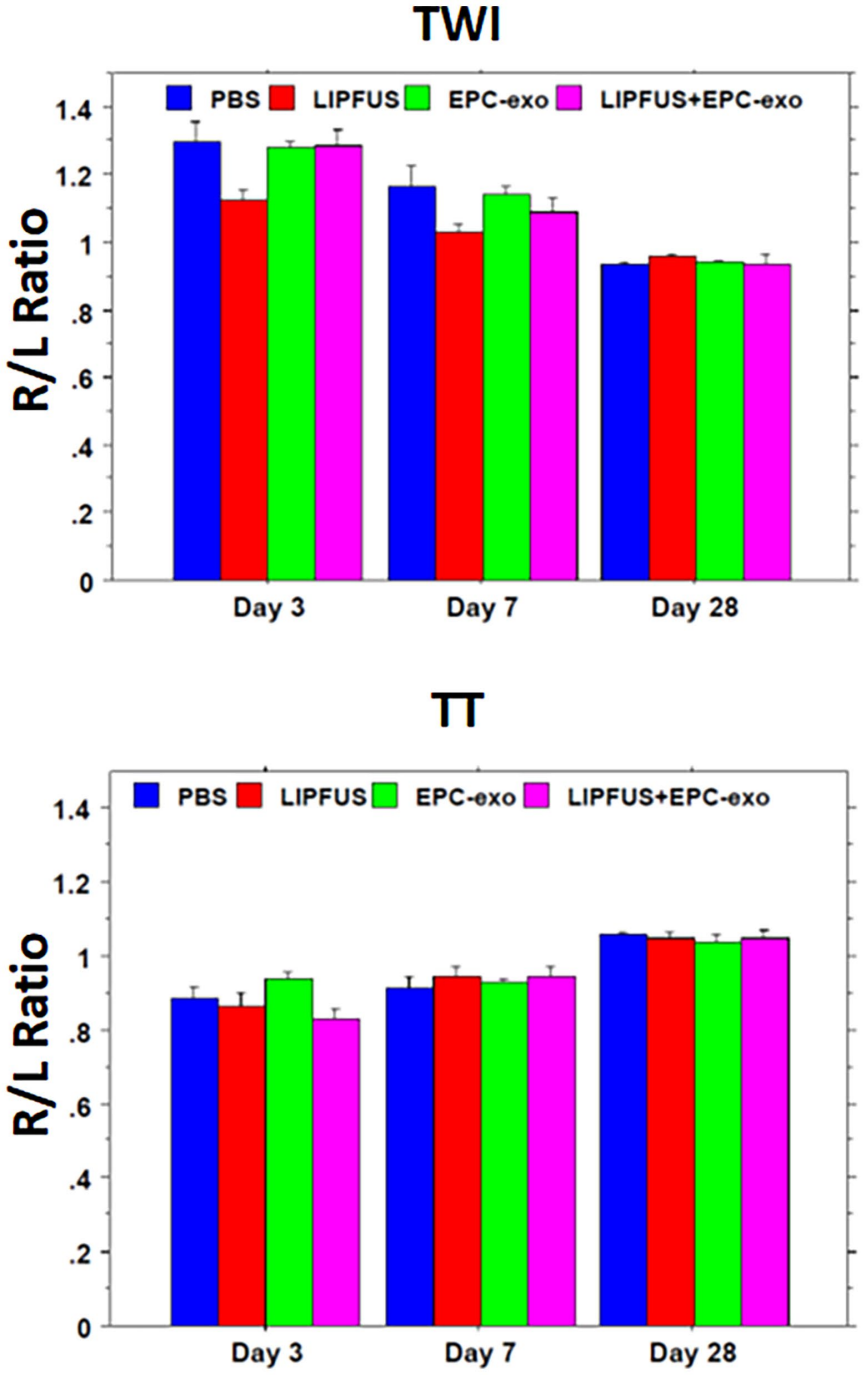
Tensor-weighted images (like diffusion-weighted images) and tensor trace (like apparent diffusion coefficient) values show no differences among the treatment groups.

Measurements of TWI and TT were conducted. TT was decreased compared to the contralateral hemisphere in all treatment groups, which were recovered by day 28. There were no significant differences among the groups on days 3, 7, and 28 (Figure 7).

### Behavioral tests correlate with stroke volume after day 3

**Figure 8:**
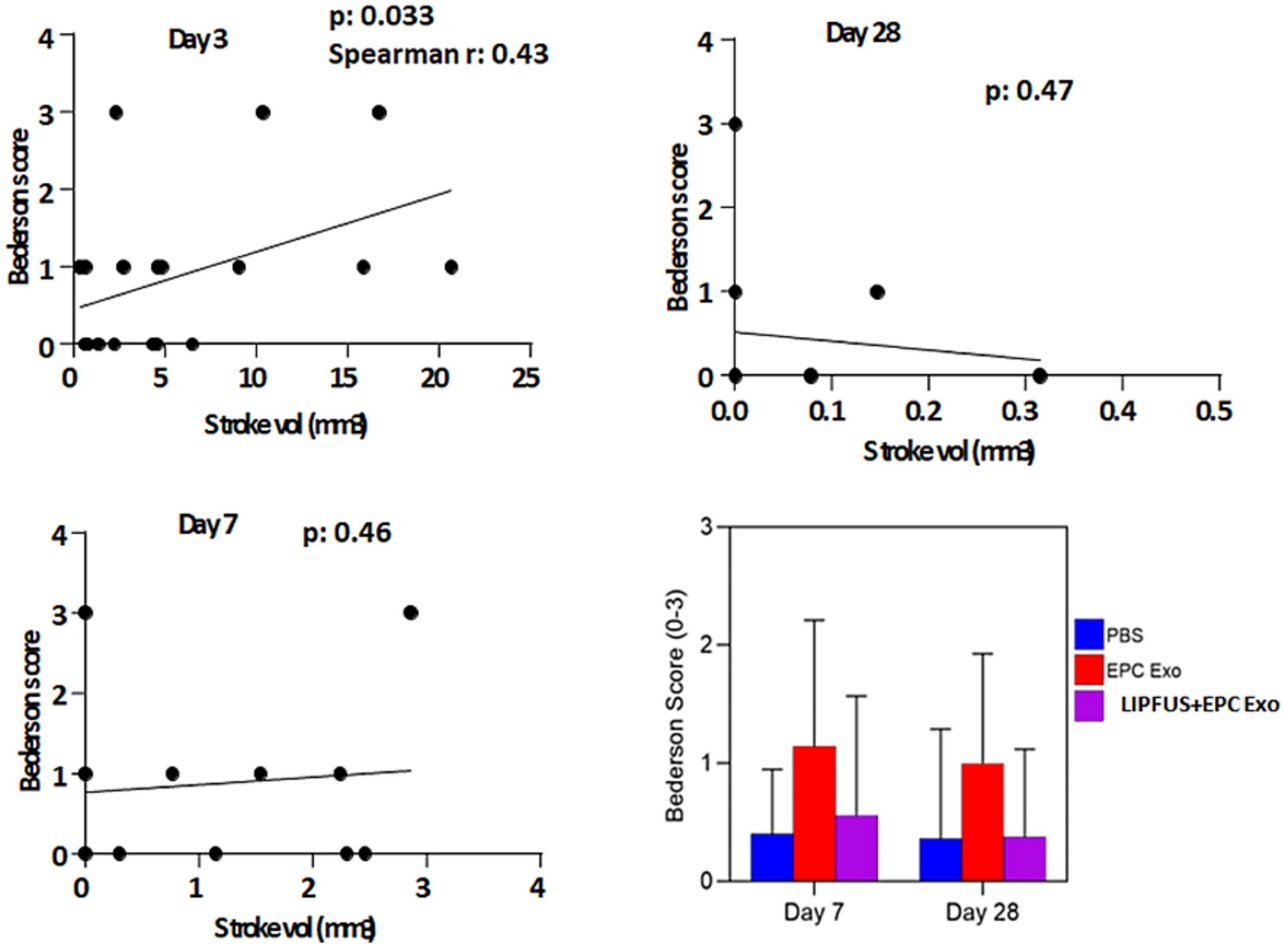
Bederson score: when all animals are together, the Bederson score is correlated with the volume of stroke size on day 3 but there is no correlation on days 7 and 8. There are also no significant differences among the treatment groups in the Benderson score on days 7 and 28 (right lower panel).

**Figure 9:**
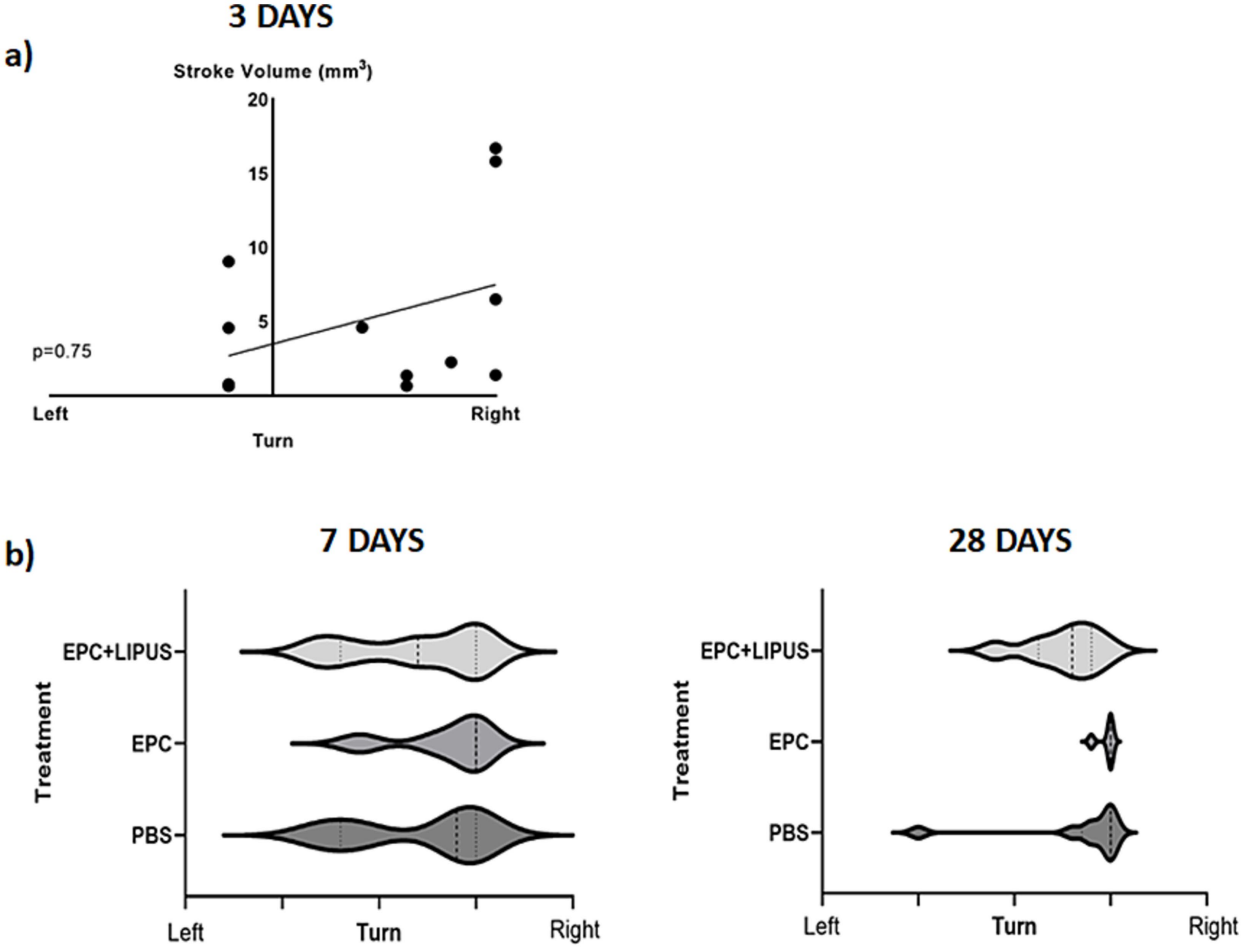
Corner test: on day 3 **(a)** animals show a significant correlation between the stroke volume and right turn. However, there are no significant differences among the treatment groups on days 7 and 28 **(b, c)**.

To determine whether LIPFUS and/or EPC exosome treatment improves the neurobehavioral function of stroke mice, we evaluated Bederson Scores (0–3) (59) of mice on day 3, day 7, and day 28. We found a significant (*p* = 0.033) correlation between the Bederson Scores of the mice and the stroke volume on day 3; larger stroke volumes demonstrated higher Bederson Scores (Figure 8, Stroke volume and Bederson Scores Day 3). We also observed mice with larger stroke volumes decreased their activity, required longer special care, and exhibited increased signs of disturbance. However, we did not observe significant differences in Bederson Scores between treatment groups (Figure 8), which might be due to the insensitivity of the Bederson Scoring system. We also conducted a Corner test (60) to determine the preference of turning the side of the mouse in the corner, but we did not find a significant correlation with stroke volume 3 days post-induction of stroke (Figure 9).

### Fluoro-Jade C staining revealed the extent of stroke damage

We utilized Fluoro-Jade C staining to evaluate the extent of stroke damage. After 28 days, Fluoro-Jade C staining revealed damaged neurons, even though MRI had not detected most of the stroke. On the other hand, we also observed a visible stroke in MRI on day 3 that wasn’t detected by Fluoro-Jade C. We found a better correlation between Fluoro-Jade C staining with other immunofluorescence staining, showing that it might be a useful tool in determining stroke areas in tissue (Supplementary Figure 4).

### Assessment of myelin integrity and structural changes

To assess the structural integrity of the brain in different treatment groups and compare the response to the treatment, we conducted LFB staining to evaluate myelin sheaths (Supplementary Figure 5). We found no difference between treatment groups, indicating the extent of brain damage after 28 days of the stroke could not be detected and differentiated using LFB staining. We also conducted Myelin Basic Protein fluorescence staining to visualize myelin structures and could not detect structural changes among the treatment groups (Supplementary Figure 6).

## Discussion

We have used four treatment groups, control or vehicle only, LIPFUS only, EPC-derived exosome only, and EPC-derived exosome along with LIPFUS to increase the delivery of exosomes. Our previous studies demonstrated that exosome delivery was increased in the brain with utilization of LIPFUS without ultrasound contrast agents (50), and we anticipated that LIPFUS would also increase the delivery of IV-administered EPC-derived exosomes to the site of stroke and would result in a better outcome in reducing the stroke volume. Our previous studies showed that 2 MPa LIPFUS with 180 bursts without nanobubbles did not cause any leakage of BBB or injury to the brain tissues (50). In this study with stroke, we decided to use 90 bursts instead of 180 bursts. We conducted biodistribution studies of radioisotope (I-131) tagged EPC-derived exosomes and detected them by using SPECT scanning. Our SPECT imaging studies showed increased accumulation of I-131 tagged EPC-exosomes to the site of stroke following sonication. As shown in our previous studies (50), applied LIPFUS (both ultrasound only group and EPC-exosomes plus ultrasound group) with our optimized parameters did not cause structural damage to the site of stroke as compared to control (vehicle or EPC-exosome treated stroke) animals. The increased accumulation of exosomes at the site of stroke following LIPFUS was probably due to transient increased BBB opening in stroke areas or transient inflammation and increased expression of adhesion molecules as we have not detected extravascular albumin in the brain parenchyma. We have also investigated whether there is an increased accumulation of bone marrow derived CD45+ cells or CD68+ cells in stroke areas that received LIPFUS, and we have not observed any significant differences in the accumulation of CD45+ or CD68+ cells indicating no structural damage in the BBB or acute inflammation following LIPFUS. Previous studies demonstrated transient sterile inflammation at the site of pulsed focal ultrasound and microbubble infusion in the brain causing transient BBB opening and extravasation of albumin was not seen after 24 h of sonication (45). There have been many investigations targeting the focal delivery of drugs or nanoparticles using focal ultrasound not only in the peripheral organs but also in the brain (47, 48); however, we are the first to show the targeted delivery of exosomes to the site of stroke using LIPFUS without causing any structural damage. We expect this technique can be used in the future for direct clinical treatment of acute-onset strokes and other brain-related events.

We have utilized different sequences of MRI and created maps for T2, rCBF, FA, TT, and TWI to assess stroke volume, ventricular volume, cerebral blood flow, and diffusion tensor parameters. Additionally, we also used MDFET sequences to identify the high signal intensity areas along the white matter tracks. Our previous study showed that most of the changes on MRI were obvious on day 14 following the administration of EPC in stroke animals (5). In this current study, we wanted to observe the early effect of EPC exosome treatment. Therefore, instead of performing MRI on day 14, we scanned our animals on days 3, and 7 and delayed it on day 28. Day 7 data indicated significant differences in the stroke volume resolution in animals that were treated with EPC-exosomes plus LIPFUS. The delayed MRI on day 28 did not depict the stroke area and was not useful to compare among the treatment groups. We also reported similar results in our recent Stroke Preclinical Assessment Network (SPAN) studies, where day 30 MRI images were not useful to determine the effects of treatment groups for lesion volume fraction (residual stroke volume) (62). In the current study, we measured lateral ventricular volumes and calculated the ratio between ipsilateral versus contra-lateral sides. MRI showed higher lateral ventricular volume on the ipsilateral side in all treatment groups on day 28; however, there were no significant differences observed among the treatment groups on days 3, 7, and 28. The increased lateral ventricular volume on the stroke side is due to the shrinking of the parenchyma/brain structures as previously reported (63, 64). We observed significantly lower shrinkage of the stroked hemisphere in the ECP exosomes-only group on day 7 scans but there were no significant changes on day 28 scans. Therefore, delayed structural MRI (for morphometry) to determine the stroke and lateral ventricular volume may not be useful for investigating the effects of different treatments in stroke. Based on the results of this study, structural MRI up to day 14 will be sufficient to determine the effect of the treatments on stroke.

We also investigated rCBF by MRI and evaluated the density of tomato lectin-positive endothelial cells in and around the stroke area in each group of animals. Despite observing a significantly higher number of tomato lectin-positive areas on immunohistochemistry in animals that were treated with EPC-exosomes plus LIPFUS, rCBF measured by MRI did not show any significant differences among the groups on both days 7 and 28. This discrepancy between MRI and immunohistochemistry might be due to nonfunctional vessels. Relative CBF measured using arterial spin labeling techniques by MRI only shows the flow to the area of interest, but tomato lection shows the presence of endothelial cells irrespective of functional blood vessel formation (65, 66). In contrast to this, the treatment with EPC-exosomes plus LIPFUS increased the proliferation or accumulation of endothelial cells at the stroke sites, which might help in reviving the dying neurons and significantly decrease stroke volume. FluoroJade C staining also proved that EPC-exosomes plus LIPFUS-treated animals qualitatively had a lower number of degenerating mature neurons.

The most striking feature we observed on MRI was the hyper-intense voxel in the region of the stroke on the MDFET sequence. This sequence is like T1W images with water suppression. These high signal intensity regions are associated with white matter tracts observed on FA maps derived from DTI. Corresponding immunohistochemistry staining depicting GFAP and myelin basic protein-positive areas indicate the activated astrocytes and white matter tracts.

Astrogliosis is a process that occurs in the brain after injury, when nearby astrocytes enlarge, proliferate, and increase their expression of GFAP (67, 68). This process can lead to the formation of glial scars, which can wrap around the injury site and limit further hemorrhage and monocyte infiltration (69). The duration of the astrocyte scar is related to the severity of the injury, and mild astrocyte activation may subside over time. Investigators have renewed their interest in astrogliosis in the management of stroke (70). Astrocyte activation may influence neural regeneration and neovascularization and improve neuronal functions following a stroke (69, 71). It has been reported that there are detrimental effects of astrogliosis (72); however, the majority of studies have indicated that astrogliosis is beneficial for stroke recovery, and targeted therapy to control astrogliosis may help stroke patients (68, 70, 72). Until now astrogliosis has been diagnosed or detected by immunohistochemistry, but more recently MRI has been used to detect astrogliosis (73, 74). It has been reported that changes in the FA are due to reactive astrocytes at the sites of traumatic brain injury (75). We have used MDFET sequences to determine the hyperintense voxels in areas that are not related to edema and fluid (76). In the current study, the high signal intensity areas on MDFET images corresponded to the site of astrogliosis determined by immunohistochemistry. FA images showed a relatively increased signal compared to the contralateral normal brain even though there were fewer white matter bundles. The high signal intensity was associated with a higher number of reactive astrocytes and scars on immunofluorescence. Further investigations are warranted to determine the astrogliosis and signal on DTI images at different stages of stroke.

We did not observe any differences among the groups in neurobehavior studies, although stroke volume determined on day 3 significantly positively correlated with Bederson’s score. Animals with larger stroke volumes preferred to turn right more often when the Corner test was employed; however, no significant differences were observed among the treatment groups. The studies showed significantly decreased stroke volume following treatment with EPC-exosomes plus LIPFUS on day 7. There was also an increased number of endothelial cells at the site of stroke as well as decreased neuronal degeneration on FluoroJade C staining, but the improvements were not reflected in the neurobehavioral studies. This discrepancy might be due to the limited sensitivity of the neurobehavior tests or the small sample size in each cohort. Our study design was to follow up to day 28 following stroke, in respect of MRI and neurobehavior. Although some previous publications pointed out the importance of follow up to 42 days for long-term follow up but recent publications from SPAN pointed out 28–30 days is acceptable to determine therapeutic effects of any experimental therapy (62).

We have targeted to have at least 10 animals per treatment group; however, due to a missing stroke on day 3 MRI and early death of animals following the stroke (probably due to a very large stroke), we ended up lower than the expected sample size. Although we have not achieved significance in statistical analysis in many of our calculated parameters, we observed a tendency of better stroke recovery in animals that were treated with ECP exosomes with or without LIPFUS.

## Conclusion

Pulsed low-intensity focal ultrasound can effectively deliver IV-administered ECP-derived exosomes to the site of stroke and stroke resolution was enhanced in the treatment groups of LIPFUS+EPC exosomes. LIPFUS can be employed to enhance the delivery of exosomes or other therapeutic probes to the site of stroke without damaging the brain structures. EPC-exosomes can serve as a therapeutic probe.

## Data Availability

The original contributions presented in the study are included in the article/Supplementary material, further inquiries can be directed to the corresponding author.

## References

[1] Wu Q Yan R Sun J. Probing the drug delivery strategies in ischemic stroke therapy. Drug Deliv. (2020) 27:1644–55. doi: 10.1080/10717544.2020.1850918, PMID: 33241704 PMC7717707

[2] Ronaldson PT Williams EI Betterton RD Stanton JA Nilles KL Davis TP. CNS drug delivery in stroke: improving therapeutic translation from the bench to the bedside. Stroke. (2024) 55:190–202. doi: 10.1161/STROKEAHA.123.043764, PMID: 38134249 PMC10752297

[3] Van Winkle JA Chen B Lei IF Pereira B Rajput PS Lyden PD. Concurrent middle cerebral artery occlusion and intra-arterial drug infusion via ipsilateral common carotid artery catheter in the rat. J Neurosci Methods. (2013) 213:63–9. doi: 10.1016/j.jneumeth.2012.12.004, PMID: 23261656 PMC3570629

[4] Sutherland BA Neuhaus AA Couch Y Balami JS DeLuca GC Hadley G. The transient intraluminal filament middle cerebral artery occlusion model as a model of endovascular thrombectomy in stroke. J Cereb Blood Flow Metab. (2016) 36:363–9. doi: 10.1177/0271678X15606722, PMID: 26661175 PMC4759672

[5] Iskander A Knight RA Zhang ZG Ewing JR Shankar A Varma NR. Intravenous administration of human umbilical cord blood-derived AC133+ endothelial progenitor cells in rat stroke model reduces infarct volume: magnetic resonance imaging and histological findings. Stem Cells Transl Med. (2013) 2:703–14. doi: 10.5966/sctm.2013-0066, PMID: 23934909 PMC3754470

[6] Jiang Q Thiffault C Kramer BC Ding GL Zhang L Nejad-Davarani SP. MRI detects brain reorganization after human umbilical tissue-derived cells (hUTC) treatment of stroke in rat. PLoS One. (2012) 7:e42845. doi: 10.1371/journal.pone.0042845, PMID: 22900057 PMC3416784

[7] Jiang Q Zhang ZG Ding GL Silver B Zhang L Meng H. MRI detects white matter reorganization after neural progenitor cell treatment of stroke. NeuroImage. (2006) 32:1080–9., PMID: 16860575 10.1016/j.neuroimage.2006.05.025

[8] Jiang Q Zhang ZG Ding GL Silver B Zhang L Meng H. Quantitative evaluation of white matter remodeling after neural progenitor cell treatment of stroke using MRI. Stroke. (2006) 37:648. doi: 10.1016/j.neuroimage.2006.05.025

[9] Jiang Q Zhang ZG Ding GL Zhang L Ewing JR Wang L. Investigation of neural progenitor cell induced angiogenesis after embolic stroke in rat using MRI. NeuroImage. (2005) 28:698–707. doi: 10.1016/j.neuroimage.2005.06.063, PMID: 16112879

[10] Kalluri R LeBleu VS. The biology, function, and biomedical applications of exosomes. Science. (2020) 367:eaau6977. doi: 10.1126/science.aau6977, PMID: 32029601 PMC7717626

[11] Rashid MH Borin TF Ara R Angara K Cai J Achyut BR. Differential in vivo biodistribution of 131I-labeled exosomes from diverse cellular origins and its implication for theranostic application. Nanomedicine. (2019) 21:102072. doi: 10.1016/j.nano.2019.102072, PMID: 31376572 PMC6814553

[12] Rashid MH Borin TF Ara R Piranlioglu R Achyut BR Korkaya H. Critical immunosuppressive effect of MDSCderived exosomes in the tumor microenvironment. Oncol Rep. (2021) 45:1171–81. doi: 10.3892/or.2021.7936, PMID: 33469683 PMC7860000

[13] Valadi H Ekstrom K Bossios A Sjostrand M Lee JJ Lotvall JO. Exosome-mediated transfer of mRNAs and micro RNAs is a novel mechanism of genetic exchange between cells. Nat Cell Biol. (2007) 9:654–9. doi: 10.1038/ncb1596, PMID: 17486113

[14] Lorentzen E Dziembowski A Lindner D Seraphin B Conti E. RNA channelling by the archaeal exosome. EMBO Rep. (2007) 8:470–6. doi: 10.1038/sj.embor.7400945, PMID: 17380186 PMC1866195

[15] Denzer K Kleijmeer MJ Heijnen HF Stoorvogel W Geuze HJ. Exosome: from internal vesicle of the multivesicular body to intercellular signaling device. J Cell Sci. (2000) 113:3365–74. doi: 10.1242/jcs.113.19.3365, PMID: 10984428

[16] Zhang ZG Chopp M. Exosomes in stroke pathogenesis and therapy. J Clin Invest. (2016) 126:1190–7. doi: 10.1172/JCI81133, PMID: 27035810 PMC4811130

[17] Li X Corbett AL Taatizadeh E Tasnim N Little JP Garnis C. Challenges and opportunities in exosome research—perspectives from biology, engineering, and cancer therapy. APL Bioeng. (2019) 3:011503. doi: 10.1063/1.5087122, PMID: 31069333 PMC6481742

[18] Colombo M Raposo G Théry C. Biogenesis, secretion, and intercellular interactions of exosomes and other extracellular vesicles. Annu Rev Cell Dev Biol. (2014) 30:255–89. doi: 10.1146/annurev-cellbio-101512-122326, PMID: 25288114

[19] Kalluri R. The biology and function of exosomes in cancer. J Clin Invest. (2016) 126:1208–15. doi: 10.1172/JCI81135, PMID: 27035812 PMC4811149

[20] ELA S Mager I Breakefield XO Wood MJ. Extracellular vesicles: biology and emerging therapeutic opportunities. Nat Rev Drug Discov. (2013) 12:347–57. doi: 10.1038/nrd3978, PMID: 23584393

[21] Lener T Gimona M Aigner L Borger V Buzas E Camussi G. Applying extracellular vesicles based therapeutics in clinical trials – an ISEV position paper. J Extracell Vesicles. (2015) 4:30087. doi: 10.3402/jev.v4.30087, PMID: 26725829 PMC4698466

[22] Jiang XC Gao JQ. Exosomes as novel bio-carriers for gene and drug delivery. Int J Pharm. (2017) 521:167–75. doi: 10.1016/j.ijpharm.2017.02.038, PMID: 28216464

[23] Alvarez-Erviti L Seow Y Yin H Betts C Lakhal S Wood MJA. Delivery of si RNA to the mouse brain by systemic injection of targeted exosomes. Nat Biotechnol. (2011) 29:341–5. doi: 10.1038/nbt.1807, PMID: 21423189

[24] El Andaloussi S Lakhal S Mager I Wood MJ. Exosomes for targeted si RNA delivery across biological barriers. Adv Drug Deliv Rev. (2013) 65:391–7. doi: 10.1016/j.addr.2012.08.008, PMID: 22921840

[25] El-Andaloussi S Lee Y Lakhal-Littleton S Li J Seow Y Gardiner C. Exosome-mediated delivery of si RNA in vitro and in vivo. Nat Protoc. (2012) 7:2112–26. doi: 10.1038/nprot.2012.131, PMID: 23154783

[26] Mashouri L Yousefi H Aref AR Am A Molaei F Alahari SK. Exosomes: composition, biogenesis, and mechanisms in cancer metastasis and drug resistance. Mol Cancer. (2019) 18:75. doi: 10.1186/s12943-019-0991-5 30940145 PMC6444571

[27] Bellavia D Raimondi L Costa V De Luca A Carina V Maglio M. Engineered exosomes: a new promise for the management of musculoskeletal diseases. Biochim Biophys Acta Gen Subj. (2018) 1862:1893–901. doi: 10.1016/j.bbagen.2018.06.003, PMID: 29885361

[28] Sterzenbach U Putz U Low L-H Silke J Tan S-S Howitt J. Engineered exosomes as vehicles for biologically active proteins. Mol Ther. (2017) 25:1269–78. doi: 10.1016/j.ymthe.2017.03.030, PMID: 28412169 PMC5474961

[29] Luan X Sansanaphongpricha K Myers I Chen H Yuan H Sun D. Engineering exosomes as refined biological nanoplatforms for drug delivery. Acta Pharmacol Sin. (2017) 38:754–63. doi: 10.1038/aps.2017.12, PMID: 28392567 PMC5520184

[30] Tian T Zhang H-X He C-P Fan S Zhu Y-L Qi C. Surface functionalized exosomes as targeted drug delivery vehicles for cerebral ischemia therapy. Biomaterials. (2018) 150:137–49. doi: 10.1016/j.biomaterials.2017.10.012, PMID: 29040874

[31] Xin H Li Y Cui Y Yang JJ Zhang ZG Chopp M. Systemic administration of exosomes released from mesenchymal stromal cells promote functional recovery and neurovascular plasticity after stroke in rats. J Cereb Blood Flow Metab. (2013) 33:1711–5. doi: 10.1038/jcbfm.2013.152, PMID: 23963371 PMC3824189

[32] Xin H Li Y Liu Z Wang X Shang X Cui Y. MiR-133b promotes neural plasticity and functional recovery after treatment of stroke with multipotent mesenchymal stromal cells in rats via transfer of exosome-enriched extracellular particles. Stem Cells. (2013) 31:2737–46. doi: 10.1002/stem.1409, PMID: 23630198 PMC3788061

[33] Webb RL Kaiser EE Scoville SL Thompson TA Fatima S Pandya C. Human neural stem cell extracellular vesicles improve tissue and functional recovery in the murine thromboembolic stroke model. Transl Stroke Res. (2018) 9:530–9. doi: 10.1007/s12975-017-0599-2, PMID: 29285679 PMC6132936

[34] Stickney Z Losacco J McDevitt S Zhang Z Lu B. Development of exosome surface display technology in living human cells. Biochem Biophys Res Commun. (2016) 472:53–9. doi: 10.1016/j.bbrc.2016.02.058, PMID: 26902116

[35] Wen SW Sceneay J Lima LG Wong CS Becker M Krumeich S. The biodistribution and immune suppressive effects of breast Cancer-derived exosomes. Cancer Res. (2016) 76:6816–27. doi: 10.1158/0008-5472.CAN-16-0868, PMID: 27760789

[36] Jang SC Kim SR Yoon YJ Park KS Kim JH Lee J. In vivo kinetic biodistribution of nano-sized outer membrane vesicles derived from bacteria. Small. (2015) 11:456–61. doi: 10.1002/smll.201401803, PMID: 25196673

[37] Watson DC Bayik D Srivatsan A Bergamaschi C Valentin A Niu G. Efficient production and enhanced tumor delivery of engineered extracellular vesicles. Biomaterials. (2016) 105:195–205. doi: 10.1016/j.biomaterials.2016.07.003, PMID: 27522254 PMC7156278

[38] Patel MM Patel BM. Crossing the blood-brain barrier: recent advances in drug delivery to the brain. CNS Drugs. (2017) 31:109–33. doi: 10.1007/s40263-016-0405-9, PMID: 28101766

[39] Li X Tsibouklis J Weng T Zhang B Yin G Feng G. Nano carriers for drug transport across the blood-brain barrier. J Drug Target. (2016) 25:17–28. doi: 10.1080/1061186X.2016.1184272, PMID: 27126681

[40] Kinoshita M McDannold N Jolesz FA Hynynen K. Targeted delivery of antibodies through the blood-brain barrier by MRI-guided focused ultrasound. Biochem Biophys Res Commun. (2006) 340:1085–90. doi: 10.1016/j.bbrc.2005.12.112, PMID: 16403441

[41] Kinoshita M McDannold N Jolesz FA Hynynen K. Noninvasive localized delivery of Herceptin to the mouse brain by MRI-guided focused ultrasound-induced blood-brain barrier disruption. Proc Natl Acad Sci USA. (2006) 103:11719–23. doi: 10.1073/pnas.0604318103, PMID: 16868082 PMC1544236

[42] Kinoshita M. Targeted drug delivery to the brain using focused ultrasound. Top Magn Reson Imaging. (2006) 17:209–15. doi: 10.1097/RMR.0b013e3180332e79, PMID: 17414078

[43] Fong SW Klaseboer E Khoo BC. Interaction of microbubbles with high intensity pulsed ultrasound. J Acoust Soc Am. (2008) 123:1784–93. doi: 10.1121/1.2836746, PMID: 18345866

[44] Hersh DS Nguyen BA Dancy JG Adapa AR Winkles JA Woodworth GF. Pulsed ultrasound expands the extracellular and perivascular spaces of the brain. Brain Res. (2016) 1646:543–50. doi: 10.1016/j.brainres.2016.06.040, PMID: 27369449 PMC5499235

[45] Kovacs ZI Kim S Jikaria N Qureshi F Milo B Lewis BK. Disrupting the blood-brain barrier by focused ultrasound induces sterile inflammation. Proc Natl Acad Sci USA. (2017) 114:E75–84. doi: 10.1073/pnas.1614777114, PMID: 27994152 PMC5224365

[46] Mesiwala AH Farrell L Wenzel HJ Silbergeld DL Crum LA Winn HR. High-intensity focused ultrasound selectively disrupts the blood-brain barrier in vivo. Ultrasound Med Biol. (2002) 28:389–400. doi: 10.1016/s0301-5629(01)00521-x 11978420

[47] Wang TY Choe JW Pu K Devulapally R Bachawal S Machtaler S. Ultrasound-guided delivery of micro RNA loaded nanoparticles into cancer. J Control Release. (2015) 203:99–108. doi: 10.1016/j.jconrel.2015.02.018, PMID: 25687306 PMC4373966

[48] Hynynen K McDannold N Vykhodtseva N Raymond S Weissleder R Jolesz FA. Focal disruption of the blood-brain barrier due to 260-kHz ultrasound bursts: a method for molecular imaging and targeted drug delivery. J Neurosurg. (2006) 105:445–54. doi: 10.3171/jns.2006.105.3.445, PMID: 16961141

[49] Zhou Y Gao XW. Effect of hydrodynamic cavitation in the tissue erosion by pulsed high-intensity focused ultrasound (pHIFU). Phys Med Biol. (2016) 61:6651–67. doi: 10.1088/0031-9155/61/18/6651, PMID: 27541633

[50] Alptekin A Khan MB Ara R Rashid MH Kong F Parvin M. Pulsed focal ultrasound as a non-invasive method to deliver exosomes in the brain/stroke. J Biomed Nanotechnol. (2021) 17:1170–83. doi: 10.1166/jbn.2021.3091, PMID: 34167630 PMC11060887

[51] Anderson SA Glod J Arbab AS Noel M Ashari P Fine HA. Noninvasive MR imaging of magnetically labeled stem cells to directly identify neovasculature in a glioma model. Blood. (2005) 105:420–5. doi: 10.1182/blood-2004-06-2222, PMID: 15331444

[52] Heissig B Werb Z Rafii S Hattori K. Role of c-kit/kit ligand signaling in regulating vasculogenesis. Thromb Haemost. (2003) 90:570–6. doi: 10.1160/TH03-03-0188, PMID: 14515175

[53] Lutz M Rosenberg M Kiessling F Eckstein V Heger T Krebs J. Local injection of stem cell factor (SCF) improves myocardial homing of systemically delivered c-kit + bone marrow-derived stem cells. Cardiovasc Res. (2008) 77:143–50. doi: 10.1093/cvr/cvm027, PMID: 18006465

[54] Tsukada S Kwon S-M Matsuda T Jung S-Y Lee J-H Lee S-H. Identification of mouse colony-forming endothelial progenitor cells for postnatal neovascularization: a novel insight highlighted by new mouse colony-forming assay. Stem Cell Res Ther. (2013) 4:20. doi: 10.1186/scrt168, PMID: 23448126 PMC3706928

[55] Steinmetz M Lucanus E Zimmer S Nickenig G Werner N. Mobilization of sca 1/flk-1 positive endothelial progenitor cells declines in apolipoprotein E-deficient mice with a high-fat diet. J Cardiol. (2015) 66:532–8. doi: 10.1016/j.jjcc.2015.02.008, PMID: 25818640

[56] Condon ET Wang JH Redmond HP. Surgical injury induces the mobilization of endothelial progenitor cells. Surgery. (2004) 135:657–61. doi: 10.1016/j.surg.2003.10.012, PMID: 15179372

[57] Janic B Guo AM Iskander ASM Varma NRS Scicli AG Arbab AS. Human cord blood-derived AC133+ progenitor cells preserve endothelial progenitor characteristics after long term in vitro expansion. PLoS One. (2010) 5:e9173. doi: 10.1371/journal.pone.0009173, PMID: 20161785 PMC2820083

[58] Zhang Z Chopp M Powers C. Temporal profile of microglial response following transient (2 h) middle cerebral artery occlusion. Brain Res. (1997) 744:189–98. doi: 10.1016/S0006-8993(96)01085-2, PMID: 9027378

[59] Bederson JB Pitts LH Germano SM Nishimura MC Davis RL Bartkowski HM. Evaluation of 2, 3, 5-triphenyltetrazolium chloride as a stain for detection and quantification of experimental cerebral infarction in rats. Stroke. (1986) 17:1304–8. doi: 10.1161/01.STR.17.6.1304, PMID: 2433817

[60] Zhang L Schallert T Zhang ZG Jiang Q Arniego P Li Q. A test for detecting long-term sensorimotor dysfunction in the mouse after focal cerebral ischemia. J Neurosci Methods. (2002) 117:207–14. doi: 10.1016/S0165-0270(02)00114-0, PMID: 12100987

[61] Saunders NR Dziegielewska KM Møllgård K Habgood MD. Markers for blood-brain barrier integrity: how appropriate is Evans blue in the twenty-first century and what are the alternatives? Front Neurosci. (2015) 9:385. doi: 10.3389/fnins.2015.00385, PMID: 26578854 PMC4624851

[62] Lyden PD Bosetti F Diniz MA Rogatko A Koenig JI Lamb J. The stroke preclinical assessment network: rationale, design, feasibility, and stage 1 results. Stroke. (2022) 53:1802–12. doi: 10.1161/STROKEAHA.121.038047, PMID: 35354299 PMC9038686

[63] Sayed MA Eldahshan W Abdelbary M Pillai B Althomali W Johnson MH. Stroke promotes the development of brain atrophy and delayed cell death in hypertensive rats. Sci Rep. (2020) 10:20233. doi: 10.1038/s41598-020-75450-6, PMID: 33214598 PMC7678843

[64] Brodtmann A Khlif MS Egorova N Veldsman M Bird LJ Werden E. Dynamic regional brain atrophy rates in the first year after ischemic stroke. Stroke. (2020) 51:e183–92. doi: 10.1161/STROKEAHA.120.030256, PMID: 32772680

[65] Robertson RT Levine ST Haynes SM Gutierrez P Baratta JL Tan Z. Use of labeled tomato lectin for imaging vasculature structures. Histochem Cell Biol. (2015) 143:225–34. doi: 10.1007/s00418-014-1301-3, PMID: 25534591

[66] Chen L Joseph G Zhang FF Nguyen H Jiang H Gotlinger KH. 20-HETE contributes to ischemia-induced angiogenesis. Vasc Pharmacol. (2016) 83:57–65. doi: 10.1016/j.vph.2016.04.002, PMID: 27084395 PMC4939128

[67] Eng LF Ghirnikar RS. GFAP and Astrogliosis. Brain Pathol. (1994) 4:229–37. doi: 10.1111/j.1750-3639.1994.tb00838.x, PMID: 7952264

[68] Sims NR Yew WP. Reactive astrogliosis in stroke: contributions of astrocytes to recovery of neurological function. Neurochem Int. (2017) 107:88–103. doi: 10.1016/j.neuint.2016.12.016, PMID: 28057555

[69] Collyer E Blanco-Suarez E. Astrocytes in stroke-induced neurodegeneration: a timeline. Front Mol Med. (2023) 3:1240862. doi: 10.3389/fmmed.2023.1240862, PMID: 39086680 PMC11285566

[70] Zhao Y Rempe DA. Targeting astrocytes for stroke therapy. Neurotherapeutics. (2010) 7:439–51. doi: 10.1016/j.nurt.2010.07.004, PMID: 20880507 PMC5084305

[71] Shen XY Gao ZK Han Y Yuan M Guo YS Bi X. Activation and role of astrocytes in ischemic stroke. Front Cell Neurosci. (2021) 15:755955. doi: 10.3389/fncel.2021.755955, PMID: 34867201 PMC8635513

[72] Barreto G White RE Ouyang Y Xu L Giffard RG. Astrocytes: targets for neuroprotection in stroke. Cent Nerv Syst Agents Med Chem. (2011) 11:164–73. doi: 10.2174/187152411796011303, PMID: 21521168 PMC3167939

[73] Lee JK Liu D Jiang D Kulikowicz E Tekes A Liu P. Fractional anisotropy from diffusion tensor imaging correlates with acute astrocyte and myelin swelling in neonatal swine models of excitotoxic and hypoxic-ischemic brain injury. J Comp Neurol. (2021) 529:2750–70. doi: 10.1002/cne.25121, PMID: 33543493 PMC8113130

[74] Garcia-Hernandez R Cerdan Cerda A Trouve Carpena A Drakesmith M Koller K Jones DK. Mapping microglia and astrocyte activation in vivo using diffusion MRI. Sci Adv. (2022) 8:eabq2923. doi: 10.1126/sciadv.abq2923, PMID: 35622913 PMC9140964

[75] Budde MD Janes L Gold E Turtzo LC Frank JA. The contribution of gliosis to diffusion tensor anisotropy and tractography following traumatic brain injury: validation in the rat using Fourier analysis of stained tissue sections. Brain. (2011) 134:2248–60. doi: 10.1093/brain/awr161, PMID: 21764818 PMC3155707

[76] Nowacki A Fiechter M Fichtner J Debove I Lachenmayer L Schüpbach M. Using MDEFT MRI sequences to target the GPi in DBS surgery. PLoS One. (2015) 10:e0137868. doi: 10.1371/journal.pone.0137868, PMID: 26366574 PMC4569189

