# Supplementary material for: Effects of low-intensity pulsed focal ultrasound-mediated delivery of endothelial progenitor-derived exosomes in tMCAo stroke
Source: Front Neurol. 2025 Apr 9;16:1543133. doi: 10.3389/fneur.2025.1543133 (PMC12014438; doi:10.3389/fneur.2025.1543133)
Supplement: Supplementary file 7 [file Supplementary_file_1.pdf]

Effects of low-intensity pulsed focal ultrasound-mediated delivery of endothelial progenitor-derived exosomes in tMCAo stroke

Supplemental Figures:

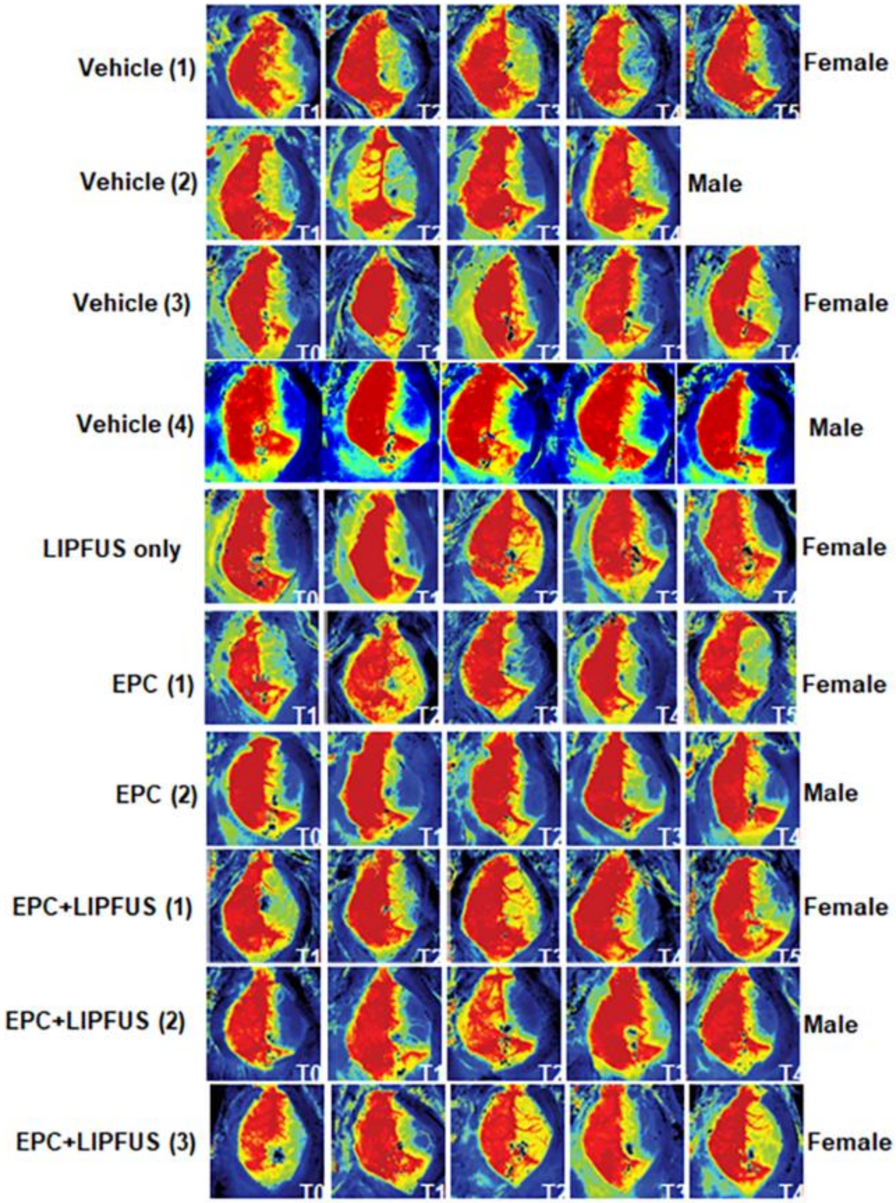

Supplemental Figure 1: Laser speckle images showing lack of blood flow during MCA occlusion.

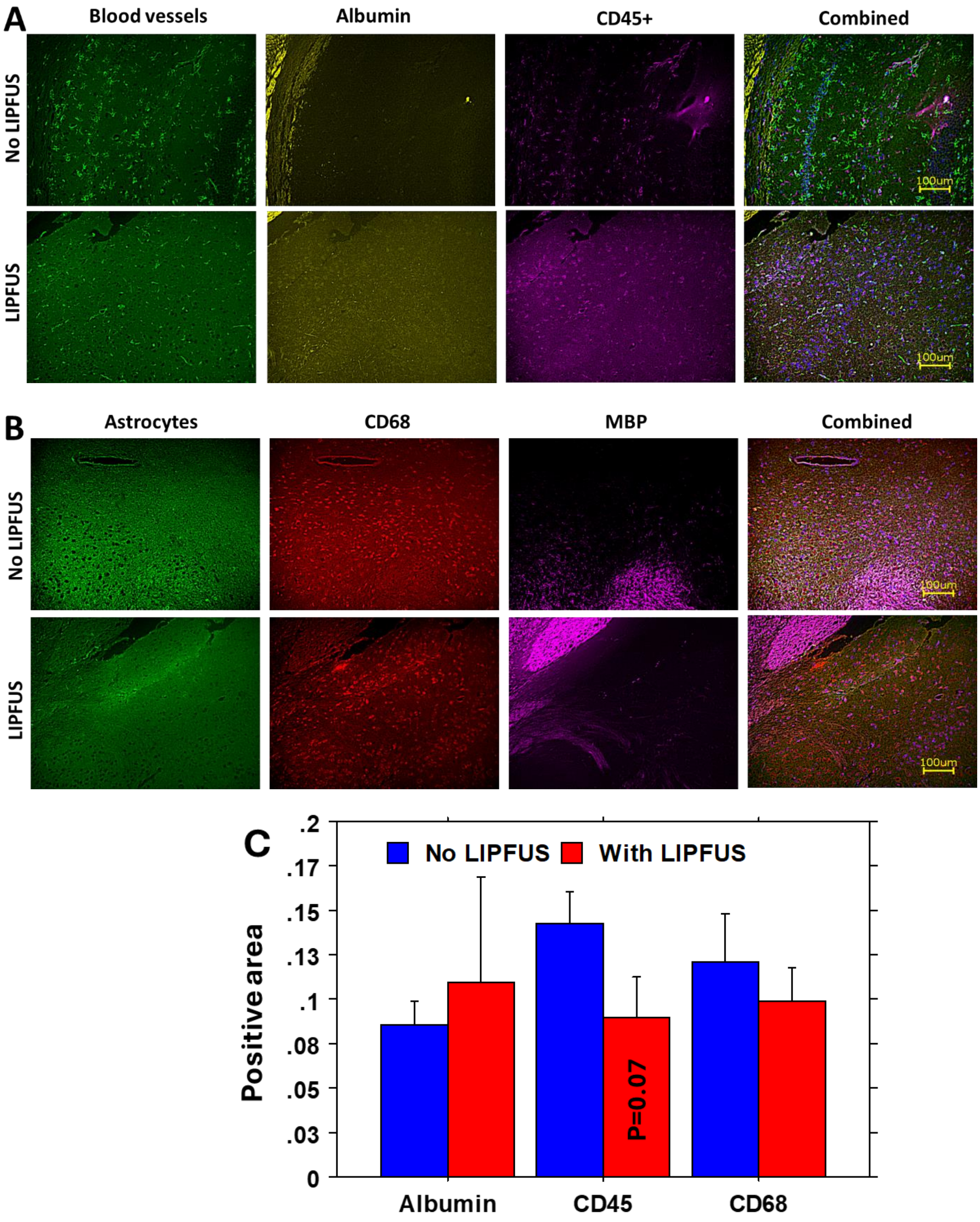

Supplemental Figure 2: IHC of different markers to indicate the effect of BBB leakage. Different markers to indicate BBB leakage and inflammatory changes (Albumin, CD45+, and CD68+ cells) were investigated. Quantitative analysis (C) showed no significant differences with or without LIPFUS.

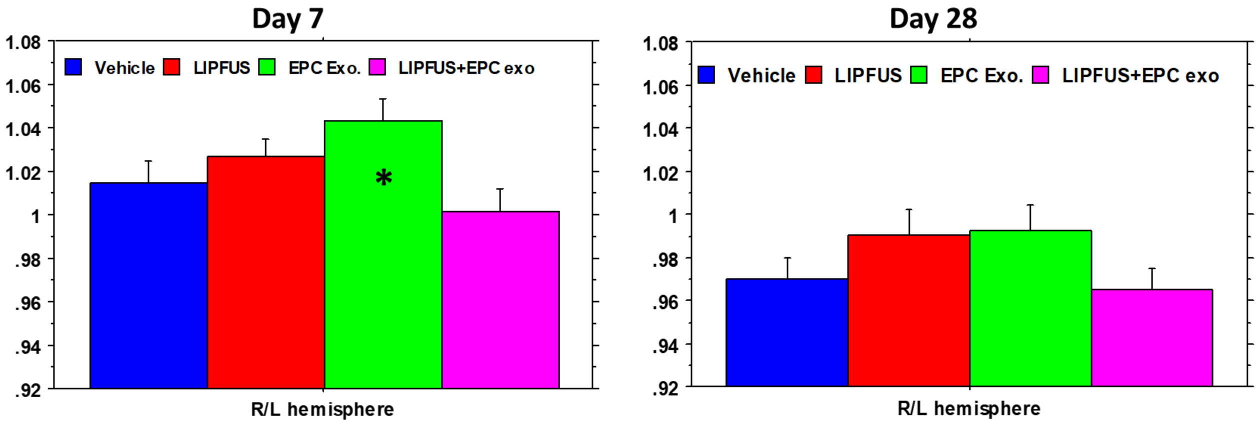

Supplemental Figure 3: Right-to-left hemisphere ratio. It is expected that the right hemisphere (stroke side) could show higher volume at early stage of stroke due to edema and the stroke side (right hemisphere) could show dramatic changes following shrinkage at later date. Therefore, the right to left hemisphere ratio was determined. No significant changes were observed among the treatment group on day 28, although EPC-exosome treated animals showed higher right to left hemisphere ratio on day 7.

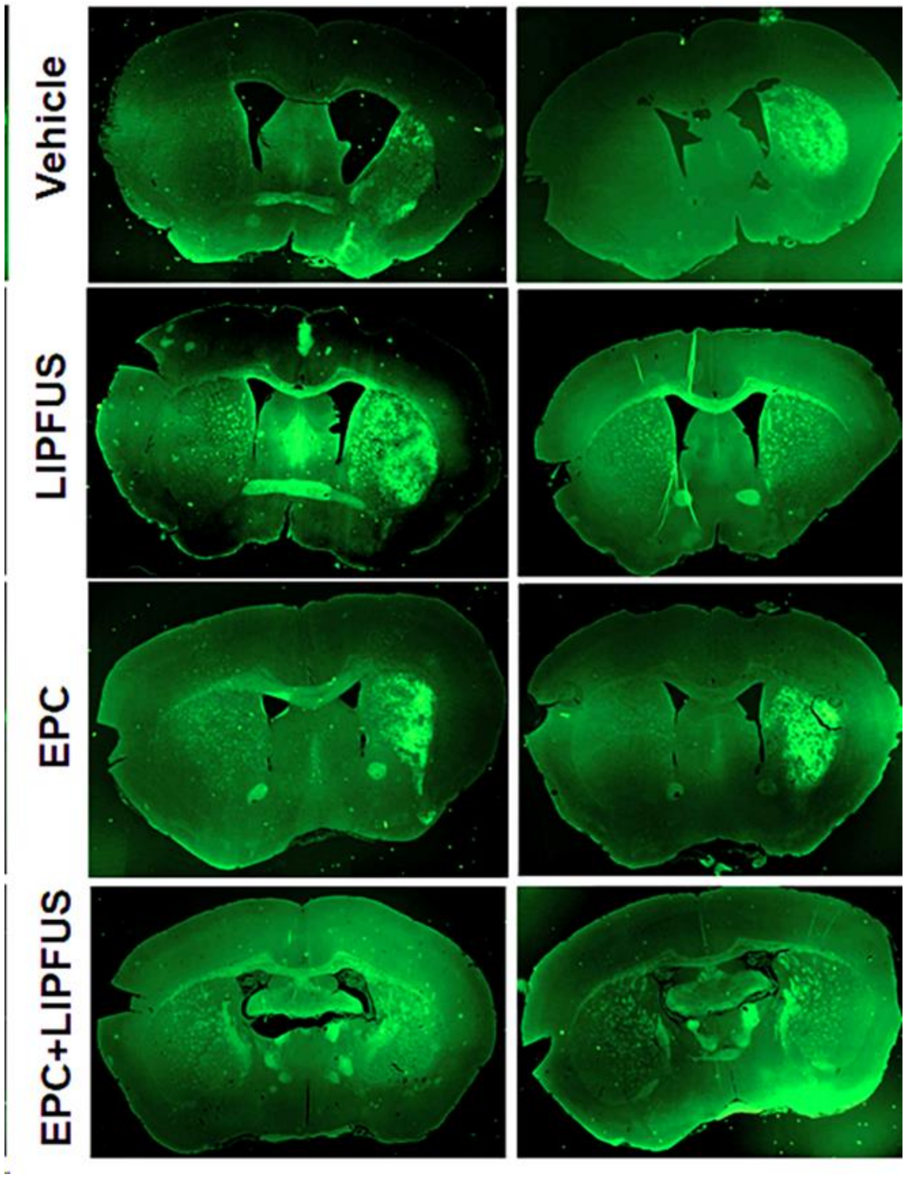

Supplemental Figure 4: Fluoro-Jade C staining. Fluoro-Jade C staining: Fluoro-Jade C staining showed a qualitatively lower number of mature neuronal damage in animals treated with LIPFUS+ ECP-exosomes.

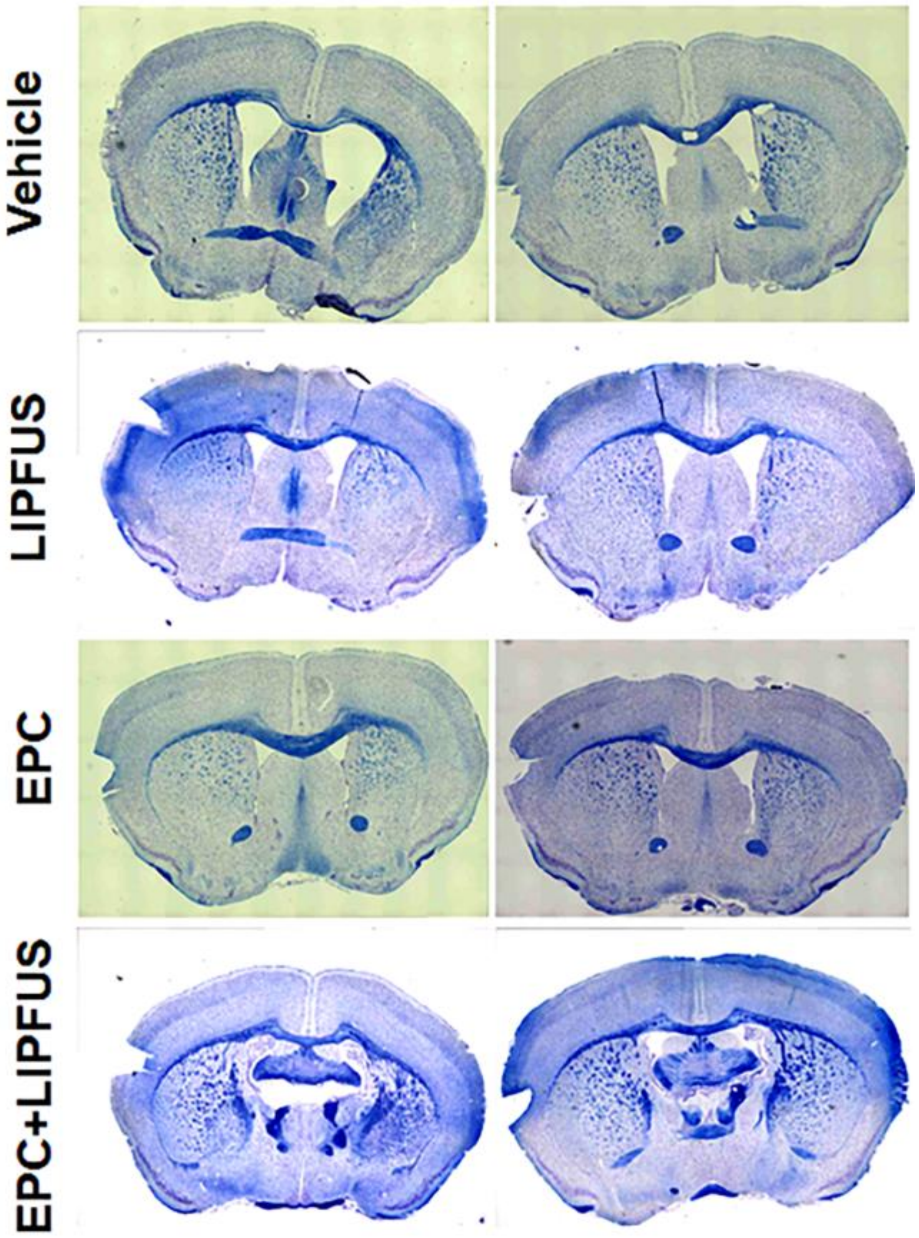

Supplemental Figure 5: Luxol Fast blue staining showing the distribution of white matter in groups of animals.

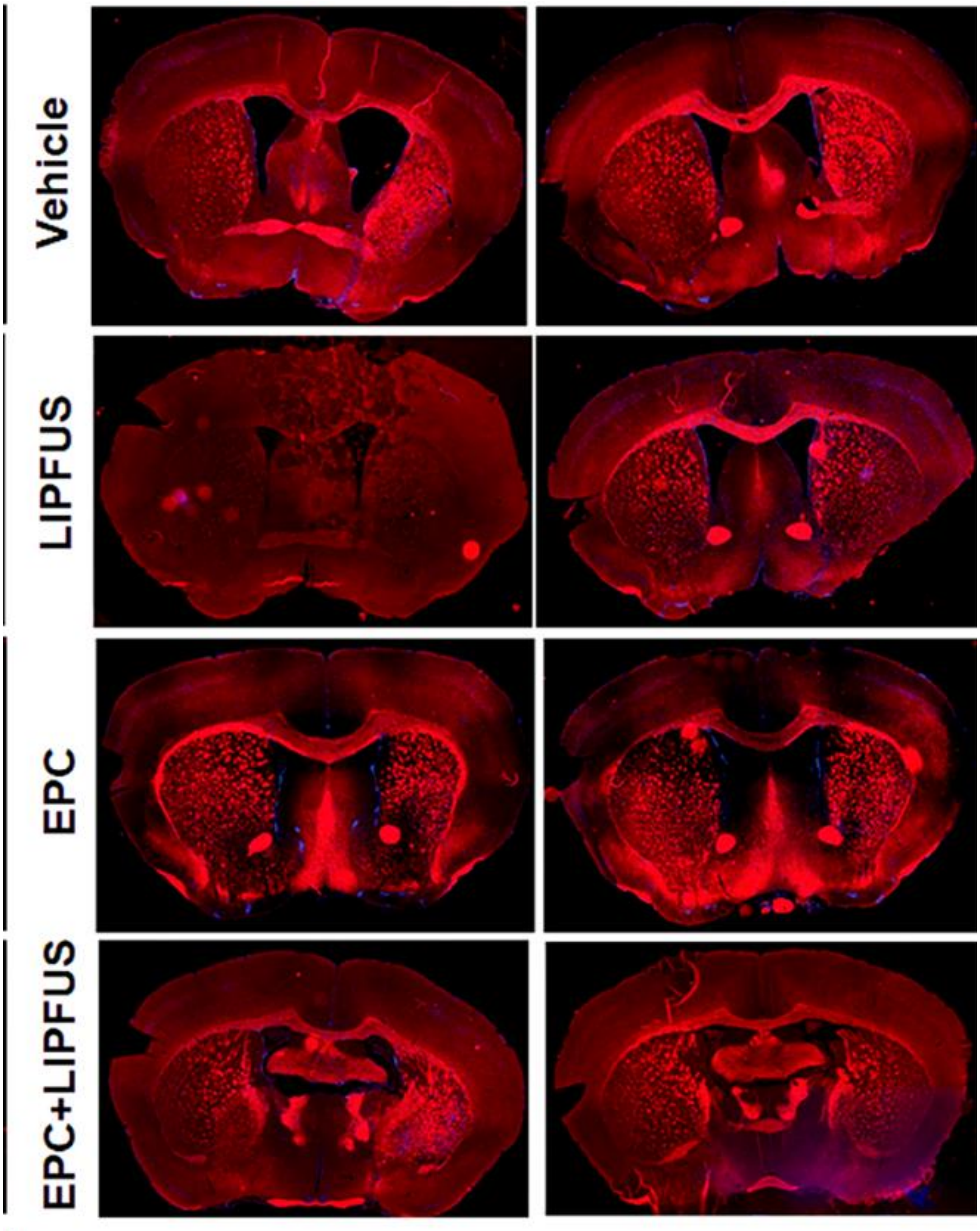

Supplemental Figure 6: Myelin Basic Protein staining showing the distribution of white matter.
